# Cancer cell discrimination and dynamic viability monitoring through wash-free bioimaging using AIEgens†

**DOI:** 10.1039/d0sc01213k

**Published:** 2020-04-30

**Authors:** Ruoyao Zhang, Guangle Niu, Qing Lu, Xiaolin Huang, Joe H. C. Chau, Ryan T. K. Kwok, Xiaoqiang Yu, Min-Hui Li, Jacky W. Y. Lam, Ben Zhong Tang

**Affiliations:** Department of Chemistry, Hong Kong Branch of Chinese National Engineering Research Center for Tissue Restoration and Reconstruction, Institute for Advanced Study, Department of Chemical and Biological Engineering, The Hong Kong University of Science and Technology Clear Water Bay Kowloon Hong Kong 999077 China chjacky@ust.hk tangbenz@ust.hk; HKUST-Shenzhen Research Institute No. 9 Yuexing 1st RD, South Area, Hi-tech Park, Nanshan Shenzhen 518057 China; Center of Bio and Micro/Nano Functional Materials, State Key Laboratory of Crystal Materials, Shandong University Jinan 250100 China; Chimie ParisTech, PSL University Paris, CNRS, Institut de Recherche de Chimie Paris 75005 Paris France min-hui.li@chimieparistech.psl.eu; Center for Aggregation-Induced Emission, SCUT-HKUST Joint Research Institute, State Key Laboratory of Luminescent Materials and Devices, South China University of Technology Guangzhou 510640 China

## Abstract

Cancer cell discrimination and cellular viability monitoring are closely related to human health. A universal and convenient fluorescence system with a dual function of wide-spectrum cancer cell discrimination and dynamic cellular viability monitoring is desperately needed, and is still extremely challenging. Herein we present a series of aggregation-induced emission luminogens (AIEgens) (denoted as IVP) which can allow accurate discrimination between cancer and normal cells and dynamic monitoring of cellular viability through mitochondria–nucleolus migration. By regulating the lengths and positions of alkyl chains in IVP molecules, we systematically studied the discrimination behavior of these AIEgens between cancer cells and normal cells and further investigated how they can migrate between the mitochondria and nucleolus based on the change of mitochondrial membrane potential (Δ*Ψ*_m_). Using **IVP-02** as a model molecule, wash-free bioimaging, excellent two-photon properties, and low cytotoxicity were demonstrated. This present work proves that these designed IVP AIEgens show great potential for cancer identification and metastasis monitoring, as well as activity evaluation and screening of drugs.

## Abstract 2

We present a series of AIEgens for cancer cell discrimination and dynamic viability monitoring.

## Graphical abstract

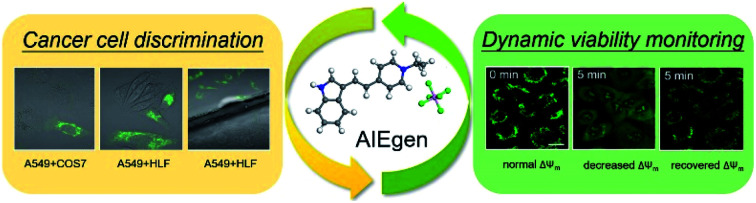

## Introduction

Cancer is one of the greatest enemies of humanity. Early detection of cancer before its metastasis is very important to increase the survival rate of patients.^1^ Different from normal cells, cancer cells overexpress some receptors, which usually serve as targets for identifying cancer cells.^2^ Currently, immunofluorescence and aptamer-based fluorescence systems are common tools for cancer detection.^3–10^ In cancer detection with immunofluorescence, antibodies conjugated with fluorescent dyes are highly specific to the overexpressed receptors, achieving high specificity to cancer cells.^3–6^ However, in the early stages of cancer, the receptors are less expressed, which increases the difficulty of early cancer detection. Moreover, the preparation of specific recognition elements against cancer cell receptors is complex and costly, and the conjugation of fluorescent dyes to antibodies sometimes affects antibody activity. In cancer detection with aptamer-based fluorescence systems, screening the aptamers specific to cancer cells and conjugation of fluorescent dyes to aptamers are also complicated processes.^7–10^ Although these specific ligands can efficiently identify cancer cell types, they are limited by the available tumour cell species, especially from unknown cancers, which makes it hard to achieve wide-spectrum cancer screening. Therefore, there is still much room for improvement of cancer detection. A universal and convenient method is strongly needed for wide-spectrum cancer cell detection.

The viability of autologous cells is closely related to human health. Monitoring cell viability is important for human health, sub-health, and disease detection. In particular, in terms of drug screening including drug development and efficacy evaluation, cell viability monitoring also plays an important role.^11^ The standard method commonly used for detecting cell viability is the MTT assay.^12^ Due to reduction by mitochondrial reductase, MTT with yellow color will turn into formazan with deep purple color. Then by measuring the absorbance of formazan at 570 nm, the cell viability can be obtained. However, MTT assay needs a long testing time, and detailed information such as the cell morphology cannot be visualized. Fluorescence microscopy is a powerful tool for *in situ* real-time detection and monitoring of biosamples *in vivo*.^13–20^ Researchers developed some fluorescent probes for cell viability detection.^21–26^ For example, calcein AM is nonfluorescent in dead cells but exhibits strong fluorescence in live cells.^24^ Fluorescein labelled annexin V is used for the detection of phosphatidylserine expression in early apoptotic cells.^25^ Propidium iodide (PI) can only stain late apoptotic and dead cells, but cannot enter live cells.^26^ However, it is hard for these probes to monitor cell viability in real time. New systems for fast and *in situ* real-time monitoring of cell viability are highly desirable but are still extremely challenging.

In response to these challenges, a variety of organic fluorophores have been developed for biosample imaging.^27–32^ Traditional aromatic and planar fluorophores have poor solubility under aqueous conditions due to the inherent hydrophobicity. Increasing the amount would lead to aggregation-caused quenching (ACQ),^33^ while in very dilute solutions, the fluorescence is too weak to be detected and easy to bleach by irradiation. Generally, a physical effect is often positively related to the amount of the added substance. As the membrane permeability and mitochondrial membrane potential (Δ*Ψ*_m_) of cancer cells is higher than those of normal cells,^34,35^ theoretically more fluorescent molecules would enter cancer cells than normal cells, providing an opportunity for cancer cell discrimination. For traditional fluorophores with the ACQ effect, fewer molecules entering normal cells lead to relatively weak fluorescence signals. However, more molecules entering cancer cells in turn lead to fluorescence decrease resulting from ACQ, thus attenuating the fluorescence signal difference between cancer and normal cells. Therefore, it is actually difficult to distinguish cancer cells from normal cells using traditional fluorophores due to the low contrast between them.

In this work, we designed and synthesized a battery of unique aggregation-induced emission luminogens (AIEgens, denoted as IVP) for cancer cell discrimination and cellular viability monitoring. Different from the fluorophores with the ACQ effect, AIEgens are highly emissive at high concentration.^33^ As cancer cells possess higher membrane permeability and Δ*Ψ*_m_ than normal cells, more AIEgens would enter cancer cells while fewer AIEgens enter normal cells. With higher concentration in cancer cells, AIEgens would emit obviously stronger fluorescence than in normal cells with fewer AIEgens. Thus, the concentration effect would amplify the fluorescence difference between cancer and normal cells, achieving cancer cell discrimination. As is known, some important positions or molecules are negatively charged inside a cell, such as mitochondria and nucleic acid in the nucleus.^36,37^ Lipophilic cations are inclined to target these negatively charged positions or molecules through electrostatic interaction.^38^ Optimizing the charge and lipophilicity of AIEgens would realize the change of dyeing position, according to variation of the cell viability. Here we designed and synthesized a series of AIEgens which could selectively stain cancer cells as well as monitor cell viability through mitochondria–nucleolus migration. Simultaneously, the relevant mechanism is carefully studied by changing the chemical structures of AIEgens.

## Results and discussion

### Design and synthesis

**Fig. 1:**
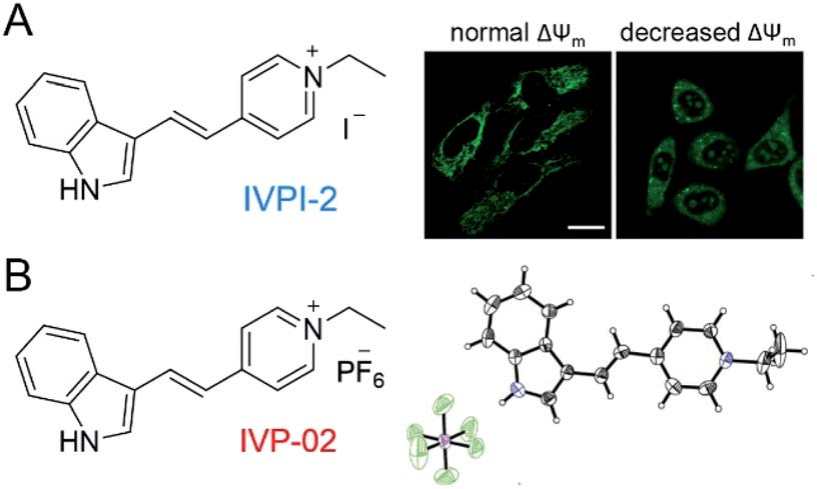
(A) Chemical structure of **IVPI-2** and the CLSM images of live HeLa cells with normal Δ*Ψ*_m_ and decreased Δ*Ψ*_m_ stained with **IVPI-2**. (B) Chemical structure of **IVP-02** and its single-molecule configuration in single crystal cells with atoms labelled in color. C, gray; H, white; N, blue; P, purple; F, green.

In our previous work shown in Fig. 1A, we found that **IVPI-2** could stain mitochondria in live cancer cells. When Δ*Ψ*_m_ decreased, it would migrate into the nucleolus.^39^ Since **IVPI-2** is target-changeable according to the change of mitochondrial physiology, we tried to modify **IVPI-2** carefully. The iodide ion is a well-known effective fluorescence quencher due to its heavy-atom effect.^40^ Consequently, the iodide ion was replaced by hexafluorophosphate and **IVP-02** was obtained as shown in Fig. 1B. The quantum yield of **IVP-02** in the solid state is 4.3%, which is about 3 times that of **IVPI-2** (1.3%). The synthetic routes to **IVP-02** are depicted in Scheme S1.† The chemical structure of **IVP-02** was fully characterized by ^1^H NMR, ^13^C NMR, ^19^F NMR and HRMS as shown in the ESI.† In addition, the structure of **IVP-02** was further confirmed by single-crystal X-ray diffraction analysis (CCDC 1986367,†Fig. 1B). The details of the experimental conditions, unit cell data and refinement data are summarized in Table S1.†

### Photophysical properties

**Fig. 2:**
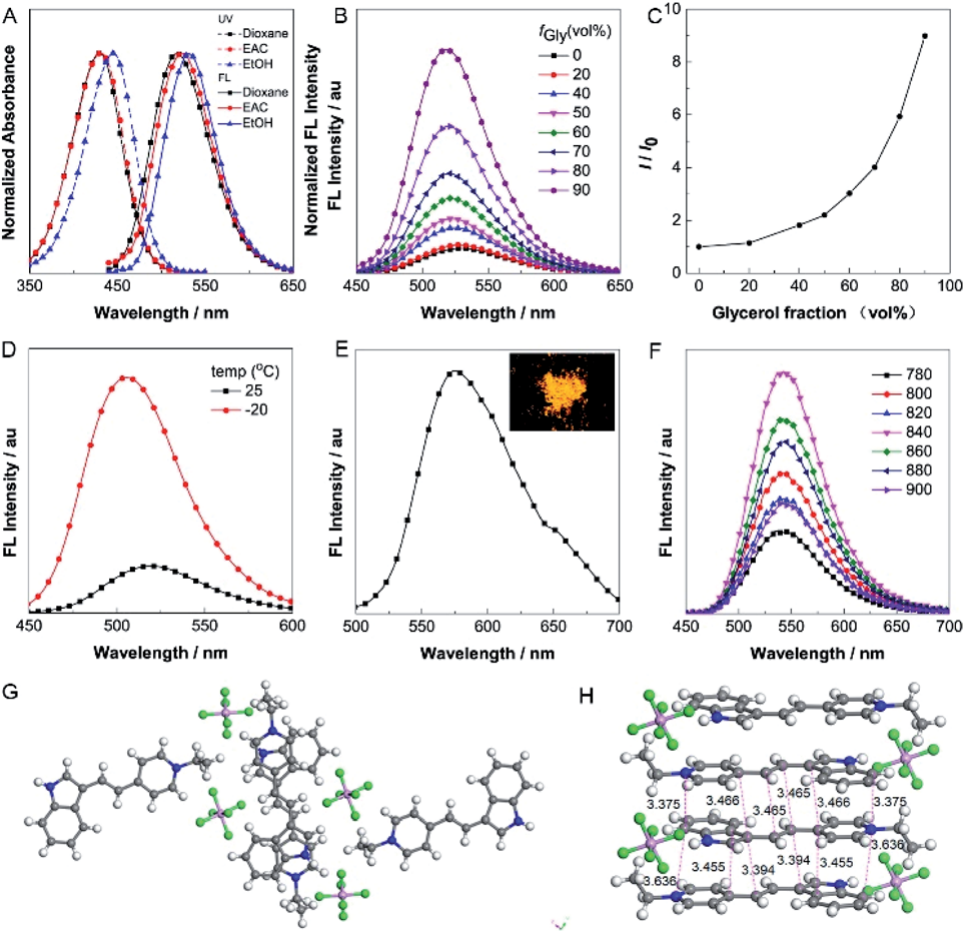
(A) Normalized UV (dashed line) and FL (solid line) spectra of **IVP-02** in different solvents. (B) FL spectra of **IVP-02** in H_2_O and H_2_O/glycerol mixtures with different glycerol fractions (*f*_Gly_). (C) Changes in the FL peak intensities (*I*) of the solutions of **IVP-02** with the glycerol content in the H_2_O/glycerol mixtures. *I*_0_ is the intensity in pure H_2_O. (D) FL spectra of **IVP-02** in the H_2_O/glycerol mixture with 90% glycerol at 25 and −20 °C. (E) FL spectrum of **IVP-02** in the solid state. *λ*_ex_ = 440 nm. Inset: fluorescence photo of **IVP-02** solid obtained under 365 nm UV irradiation using a handheld UV lamp. (F) TPEF spectra of **IVP-02** in DMSO excited by 780, 800, 820, 840, 860, 880, and 900 nm, respectively. Concentration: 10 μM. The top view (G) and side view (H) of the crystal structure of **IVP-02**.

The absorption and one-photon excited fluorescence (FL) spectra of **IVP-02** in different solvents are shown in Fig. 2A. **IVP-02** showed strong absorbance from 400 to 450 nm and intense emission from 500 to 550 nm. **IVP-02** possesses a distinct donor–π–acceptor structure. It showed a bathochromic shift in the FL spectra with the increase of solvent polarity, due to intramolecular charge transfer effect. Addition of THF and EtOH to the solution of **IVP-02** in water failed to make the dye molecules aggregate probably due to their amphiphilic nature. Similar results were reported by others.^41–43^ Then the emission of **IVP-02** in the increasingly viscous environment and in the solid state was carefully measured to study whether **IVP-02** possessed AIE activity. In Fig. 2B and C, **IVP-02** shows weak emission in pure water solution. With the increase of the glycerol volume content accompanied by the increasing viscosity, the FL intensity increased gradually. In addition to a water/glycerol system, similar experiments were carried out in the MeOH/glycerol system in Fig. S1A and B.† The results also showed that **IVP-02** was highly emissive in a high viscosity environment. Moreover, when the solution temperature decreased from 25 °C to −20 °C, the FL intensity also increased obviously as shown in Fig. 2D and S1C.† These phenomena occur because high viscosity and low temperature could hamper intramolecular motion, leading to the closure of the nonradiative decay channel and thus enhanced FL emission.^33^ Furthermore, we added some RNA in a PBS solution of **IVP-02**. **IVP-02** showed weak emission in PBS solution, but with the increase of RNA concentration, the FL intensity increased obviously as shown in Fig. S1D.† Based on the calculation results in Fig. 9, **IVP-02** located in the minor grooves of RNA, where the intramolecular motion of **IVP-02** was also hampered. These results indicated that the restriction of intramolecular motion (RIM) is the main reason that makes the dye highly emissive, which is also the luminescence mechanism of AIEgens. **IVP-02** also exhibited strong fluorescence around 575 nm in the solid state and its powder emitted bright yellow light (Fig. 2E). Therefore, based on the above results, **IVP-02** possesses AIE activity.

**IVP-02** shows weak emission in aqueous solution, but emits strong fluorescence under high-viscosity conditions; thus it is greatly favourable for wash-free bioimaging. In addition, **IVP-02** emits redder fluorescence in the solid state than in solution. To confirm the mechanism of the red shift in the solid state of **IVP-02**, its crystal is analyzed as shown in Fig. 2G and H. The molecules of **IVP-02** are anti-parallelly stacked and form multimers in the crystalline state. The short intermolecular stacking distances of the multimers are 3.636 Å, 3.455 Å, 3.394 Å, 3.375 Å, 3.466 Å, and 3.465 Å, indicating strong intermolecular interactions inside the multimers. So the red-shift emission in the solid state should be attributed to the intermolecular π–π interactions induced by the short contact between the molecules. Generally, organic dyes with a donor–acceptor structure exhibit good two-photon absorption (TPA) and two-photon excited fluorescence (TPEF).^44^ The TPEF spectra of **IVP-02** excited at different pulse wavelengths (780–900 nm) in DMSO are shown in Fig. 2F. Using fluorescein as the standard, the two-photon absorption cross section (*δ*) of **IVP-02** was calculated and is shown in Table S2.† The highest *δ* was 287 GM excited at 800 nm. Such a high *δ* value is beneficial for two-photon imaging in live cells and deep tissues.

### Cancer cell discrimination

**Fig. 3:**
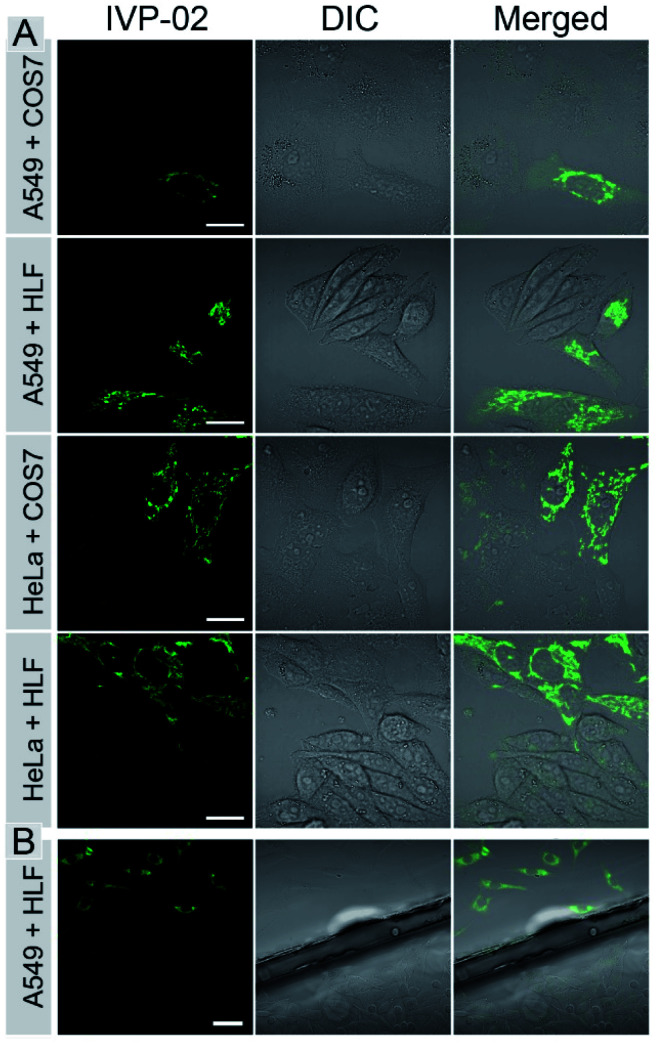
CLSM images of live cancer cells (A549 and HeLa) co-cultured with normal cells (COS7 and HLF) (A) and the images of A549 and HLF cells seeded on different cover glasses (B) stained with 2 μM **IVP-02** for 30 min, respectively. *λ*_ex_ = 488 nm, *λ*_em_ = 500–650 nm. Scale bar = 20 μm (A); scale bar = 50 μm (B).

The bioimaging properties of **IVP-02** in live cells were investigated by confocal laser scanning microscopy (CLSM). Cancer cells (A549 and HeLa) and normal cells (COS7 and HLF) were stained with **IVP-02**. In Fig. S2,† the fluorescence intensity in normal cells was much weaker than that in cancer cells under the same staining and imaging conditions. So **IVP-02** has a high potential to differentiate cancer and normal cells. To verify this speculation, cancer and normal cells were co-cultured and stained with **IVP-02**. In Fig. 3A, it could be seen that only cancer cells (A549 and HeLa) were highly illuminated, while normal cells (COS7 and HLF) showed almost no fluorescence. To precisely confirm the selectivity of **IVP-02** to cancer cells, cancer cells A549 and HeLa and normal cells COS7 and HLF were seeded on different cover glasses, respectively. Then two cover glasses with cancer and normal cells, respectively, were placed in the same dish and stained with **IVP-02** at the same time. In Fig. 3B and S3,† only cancer cells (A549 and HeLa) on the upper glass are illuminated, while normal cells (COS7 and HLF) on the lower glass are not illuminated. These results indicated that **IVP-02** could selectively differentiate cancer cells from normal cells.

In the above pictures, filamentous structures in the cytoplasm of cancer cells were observed, which are the typical morphology of mitochondria. Then co-staining experiments with the commercial mitochondrial probe MitoTracker Deep Red FM (MTDR) were carried out (Fig. S4†). The co-localization coefficient of **IVP-02** and MTDR was around 0.9, demonstrating the localization of **IVP-02** in mitochondria in cancer cells.

### Mechanism study

Then we carefully studied why **IVP-02** could selectively stain cancer cells over normal cells. Some previous work indicated the possible reason that the Δ*Ψ*_m_ of cancer cells is much higher than that of normal cells.^45^ However, it is a fact that some commercial mitochondrial probes and reported ones, also sensitive to Δ*Ψ*_m_, can stain both cancer and normal cells.^46^ So Δ*Ψ*_m_ is not the only reason that enables dyes to distinguish cancer and normal cells. The chemical structure of the dye itself also plays an important role.

**Fig. 4:**
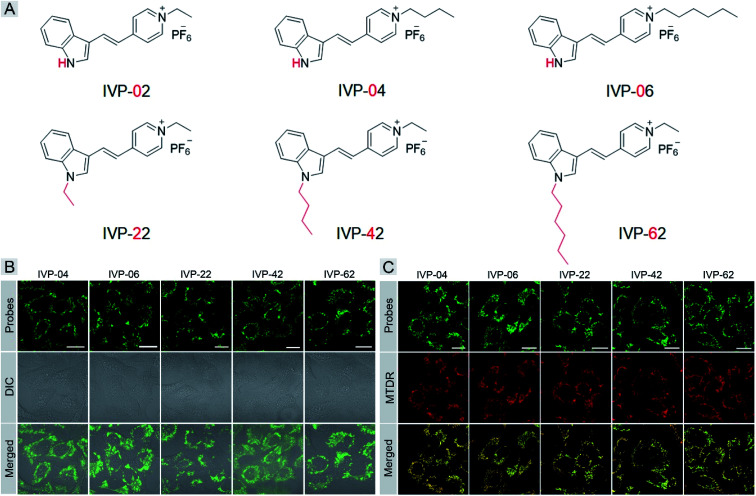
(A) Chemical structures of **IVP-02**, **04**, **06**, **22**, **42**, and **62**. (B) CLSM images of live A549 cells stained with 2 μM **IVP-04**, **06**, **22**, **42**, and **62** for 30 min, respectively. *λ*_ex_ = 488 nm, *λ*_em_ = 500–650 nm. (C) CLSM images of A549 cells stained with 2 μM **IVP-04**, **06**, **22**, **42**, and **62** and 0.2 μM MTDR, respectively. **IVP-04**, **06**, **22**, **42**, and **62**: *λ*_ex_ = 488 nm, *λ*_em_ = 500–650 nm; MTDR: *λ*_ex_ = 640 nm, *λ*_em_ = 650–700 nm. Scale bar = 20 μm.

We speculated that the membrane permeability of the probe was a key factor in its selectivity to cancer cells, since the plasma membrane of cancer cells was reported to be more permeable than that of normal cells. Then we tried to modify **IVP-02** by lengthening the alkyl chain on the pyridine salt side and indole side, respectively, to tune the membrane permeability.^47^ Five new molecules **IVP-04**, **IVP-06**, **IVP-22**, **IVP-42**, and **IVP-62** were obtained as shown in Fig. 4A. The synthetic routes to these new IVP molecules are depicted in Scheme S1.† Their chemical structures were fully characterized by ^1^H NMR, ^13^C NMR, and ^19^F NMR as shown in the ESI.† The FL spectra of **IVP-04**, **IVP-06**, **IVP-22**, **IVP-42**, and **IVP-62** in water and glycerol are shown in Fig. S5.† It could be seen that all of the five IVP molecules showed weak emission in aqueous solution, but exhibited strong fluorescence in glycerol with high viscosity. Thus they could be used in wash-free bioimaging. Then cancer cells A549 were stained with the five new molecules separately. In Fig. 4B, the fluorescence pattern showed that all these molecules stained mitochondria. The co-staining experiments with MTDR also confirmed their location in mitochondria in A549 cells in Fig. 4C. In addition to A549 cells, the same experiments were also carried out in HeLa cells (Fig. S6 and S7†). The results were similar to those in A549 cells. So **IVP-04**, **06**, **22**, **42**, and **62** can selectively stain mitochondria in cancer cells.

**Fig. 5:**
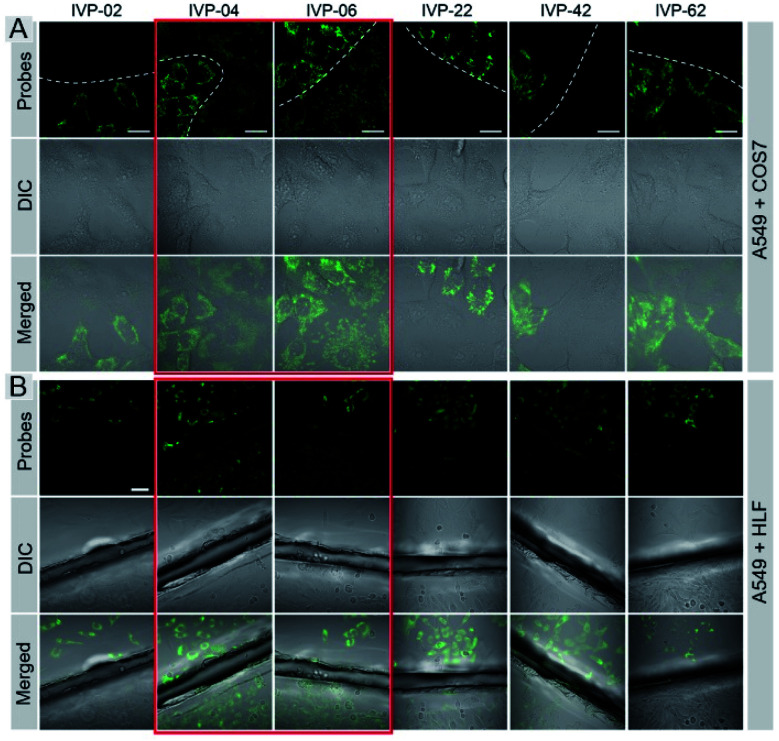
CLSM images of live cancer cells (A549) co-cultured with normal cells (COS7) (A) and the images of A549 and HLF cells seeded on different cover glasses (B) stained with 2 μM **IVP-02**, **04**, **06**, **22**, **42**, and **62** for 30 min, respectively (the first column in (B) is the same as Fig. 3B). *λ*_ex_ = 488 nm, *λ*_em_ = 500–650 nm. Scale bar = 20 μm (A); scale bar = 50 μm (B).

Cancer cells A549 co-cultured with normal cells COS7 were stained with these molecules separately. As shown in Fig. 5A, **IVP-02**, **22**, **42**, and **62** can only stain A549 cells, indicating that they can distinctly differentiate cancer and normal cells. Intriguingly, **IVP-04** and **06** can stain both A549 and COS7 cells, which means that they cannot distinguish cancer and normal cells. Then HeLa cells co-cultured with COS7 cells (Fig. S8†) and A549 cells co-cultured with HLF cells (Fig. S9†) were also stained with these molecules; the results were similar to those in A549 cells co-cultured with COS7 cells. In addition to co-culturing, A549 cells and HLF cells were also seeded on different cover glasses and stained with these molecules. The imaging results (Fig. 5B) were also similar to the co-culturing results in which **IVP-04** and **IVP-06** could stain both A549 and HLF cells, while the other four molecules only stain A549 cells. Based on the imaging results above, preliminary conclusions could be drawn that the selectivity of these IVP molecules to cancer cells is also based on the length of the alkyl chain on the pyridinium salt side. Lengthening the alkyl chain on the pyridinium salt side will eliminate the selectivity to cancer cells.

**Fig. 6:**
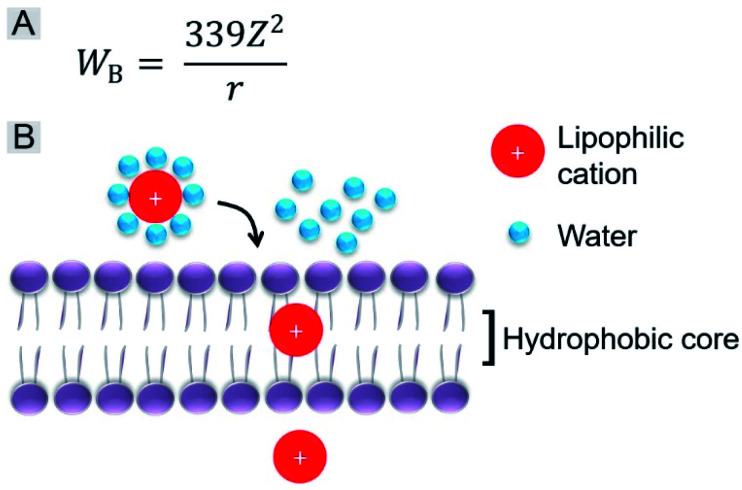
(A) Equation of Born energy. (B) Schematic diagram of a lipophilic cation passing through the membrane.

We further investigated the role of the alkyl chain on the pyridinium salt side. The way in which these IVP molecules enter the cells was first investigated. Cancer cells A549 were incubated with the IVP molecules at 4 °C for 20 min. In Fig. S10,† at low temperature, obvious fluorescence signals inside the cells could still be observed. This result indicated that these IVP molecules entered the cell by diffusion. In some reported studies, researchers found that lipophilic cations cross membranes very well.^38,48^ The activation energy for moving a lipophilic cation from the aqueous medium to the hydrophobic core of a membrane is mainly from electrostatic interactions. The main electrostatic energy component, Born energy (*W*_B_, Fig. 6A), is due to the enthalpy input required to remove water molecules from the cation upon transfer from the aqueous environment to the lipid core of the membrane.^48^ With lower *W*_B_, the lipophilic cation can pass through the membrane more easily. The Born energy is given by the equation in Fig. 6A, in which *Z* is the cation charge and *r* is the ionic radius. From the equation, *W*_B_ is inversely proportional to the ionic radius. For these IVP molecules, the ionic radius is the average distance from the molecule charge to the water molecules around. Fig. 6B shows a schematic diagram of a lipophilic cation passing through the membrane. Removing water molecules around the lipophilic cation is the first step. For the lipophilic cation, the larger the ionic radius, the weaker the interaction between the cation and water molecule and the lower the *W*_B_; as a result, the molecule passes more easily through the membrane.

Regarding the chemical structures of these IVP molecules, the pyridinium side of all these molecules is positively charged. This side is more hydrophilic, meaning that more water molecules are enriched on this side, so this side determines the ionic radius. Moreover, the similarity of **IVP-02**, **22**, **42**, and **62** on the pyridinium side is the 2-carbon alkyl chain. The difference between **IVP-02**, **22**, **42**, and **62** and **IVP-04** and **06** on the pyridinium side is the length of the alkyl chain. **IVP-04** and **06** have longer alkyl chains on the pyridinium side, so that their ionic radius is larger than that of **IVP-02**, **22**, **42**, and **62**, implying that the interaction between the **IVP-04** and **06** and water molecules is weaker. So the Born energy values of **IVP-04** and **06** are lower than those of **IVP-02**, **22**, **42**, and **62**. As such, they pass through the membrane more easily. When staining normal cells, **IVP-04** and **06** more easily penetrate cytomembrane than **IVP-02**, **22**, **42**, and **62**.

### Cellular viability monitoring

**Fig. 7:**
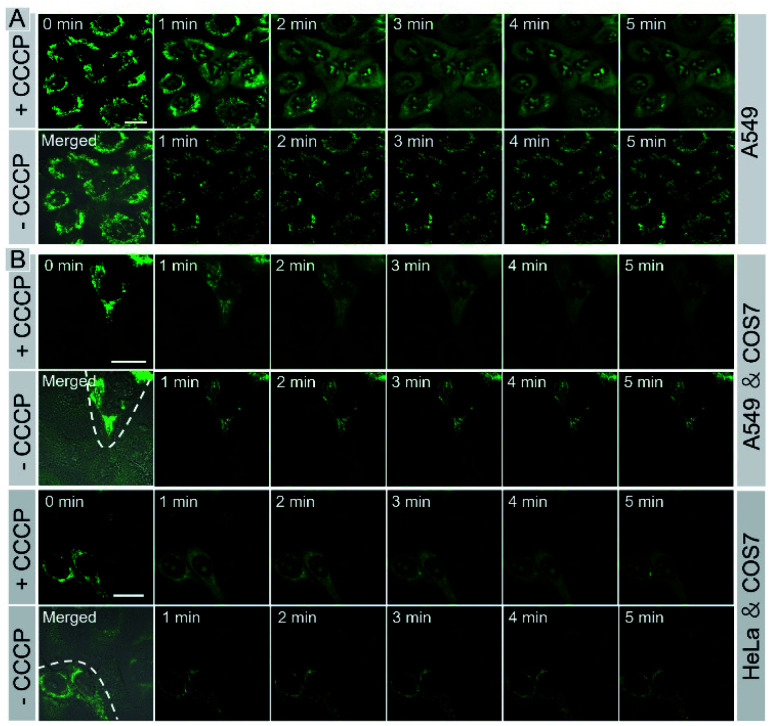
Live A549 cells (A), live A549 co-cultured with COS 7 cells, and live HeLa co-cultured with COS7 cells (B) were pre-stained with 2 μM **IVP-02** for 30 min and treated with 20 μM CCCP for 5 min. Then CCCP was removed and fresh culture medium was added for another 5 min. *λ*_ex_ = 488 nm, *λ*_em_ = 500–650 nm. Scale bar = 20 μm.

Monitoring cell viability is a highly valuable task for fundamental research in biology, pathology, and medicine.^11^ We investigated whether **IVP-02** could monitor cell viability. Δ*Ψ*_m_ is a very important indicator to characterize cell viability.^49–51^ With the loss of cell viability, Δ*Ψ*_m_ would decrease, while with the recovery of cell viability, Δ*Ψ*_m_ would also recover to the normal level. Hence visualizing the change of Δ*Ψ*_m_ is an effective way to monitor cell viability. Carbonyl cyanide *m*-chlorophenyl hydrazone (CCCP) is a type of protonophore and can cause rapid acidification of mitochondria by decreasing Δ*Ψ*_m_.^52^ Cancer cells A549 were pre-stained with **IVP-02** for 30 min. In Fig. 7A, in the beginning, **IVP-02** stained mitochondria clearly. With the addition of CCCP, the fluorescence intensity in mitochondria decreased, while that in the nucleolus increased, meaning that **IVP-02** was released from mitochondria and migrated to the nucleolus. Then after removal of CCCP with the recovery of Δ*Ψ*_m_, the fluorescence in the nucleolus disappeared, while the fluorescence in mitochondria recovered gradually, indicating that **IVP-02** migrated back to the mitochondria. In addition to A549 cells, similar results were also observed in HeLa cells as shown in Fig. S11.† Hydrogen peroxide (H_2_O_2_) can inhibit the oxidative respiratory chain which would cause the loss of cell viability.^53^ Then live A549 and HeLa cells were pre-stained with **IVP-02** and treated with 10 mM H_2_O_2_. In Fig. S12,† it can be seen that after adding H_2_O_2_ with the cell viability decreasing, strong fluorescence was observed in the nucleolus. These results indicated that **IVP-02** could monitor cancer cell viability through mitochondria–nucleolus migration.

To test whether **IVP-02** could selectively stain cancer cells and monitor their viability, cancer cells and normal cells were co-cultured and stained with **IVP-02**. As we predicted, only the mitochondrial morphology in A549 cells is clearly shown in Fig. 7B. After CCCP was added, **IVP-02** was released from mitochondria and migrated to the nucleolus. After CCCP was removed, the fluorescence in the nucleolus disappeared, while the fluorescence in mitochondria recovered gradually. In the whole process, little fluorescence was observed in normal cells COS7. The same experiments were also carried out in HeLa cells co-cultured with COS7 cells as shown in Fig. 7B and similar phenomena were observed. These results demonstrated that **IVP-02** could selectively stain cancer cells and monitor their viability through mitochondria–nucleolus migration in coexisting cancer cells and normal cells.

**Fig. 8:**
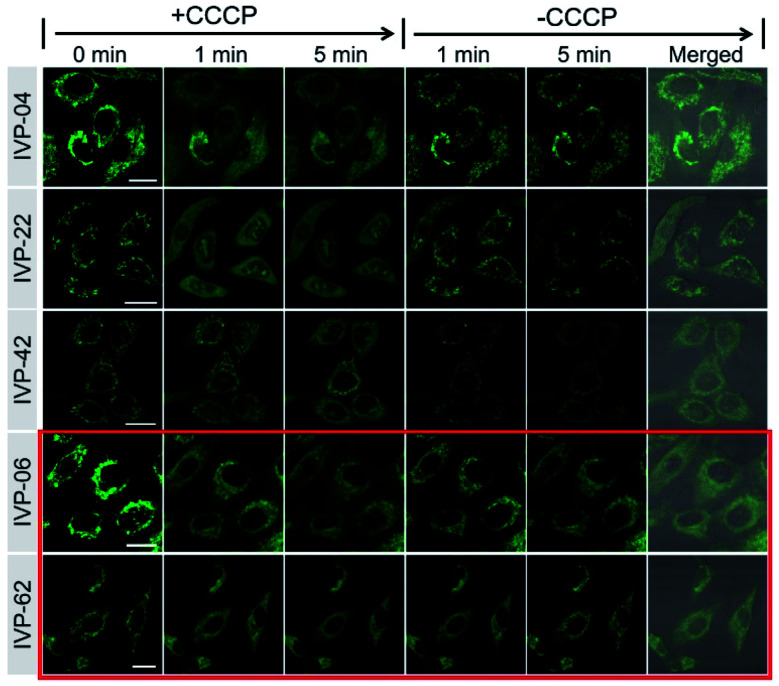
Live A549 cells were pre-stained with 2 μM **IVP-04**, **22**, **42**, **06**, and **62** for 30 min and treated with 20 μM CCCP for 5 min. Then CCCP was removed and fresh culture medium was added for another 5 min. *λ*_ex_ = 488 nm, *λ*_em_ = 500–650 nm. Scale bar = 20 μm.

In addition to **IVP-02**, we further investigated whether the other IVP molecules could monitor the viability of cancer cells through mitochondria–nucleolus migration. A549 cells were pre-stained with **IVP-04**, **22**, **42**, **06**, and **62** separately, and then CCCP was added. In Fig. 8 and S13–S17,† after addition of CCCP with the Δ*Ψ*_m_ decreasing, only **IVP-04**, **22**, and **42** could enter the nucleus and stain the nucleolus, while **IVP-06** and **62** could not. After removal of CCCP with the recovery of Δ*Ψ*_m_, **IVP-04**, **22**, and **42** could migrate back to the mitochondria. To eliminate cell interference, the same experiments were performed in HeLa cells as shown in Fig. S18–S23.† Consistent with that in A549 cells, **IVP-02**, **04**, **22**, and **42** could stain the nucleolus while **IVP-06** and **62** could not. This means **IVP-02**, **04**, **22**, and **42** can monitor the viability of cancer cells through mitochondria–nucleolus migration, while **IVP-06** and **62** cannot.

**Fig. 9:**
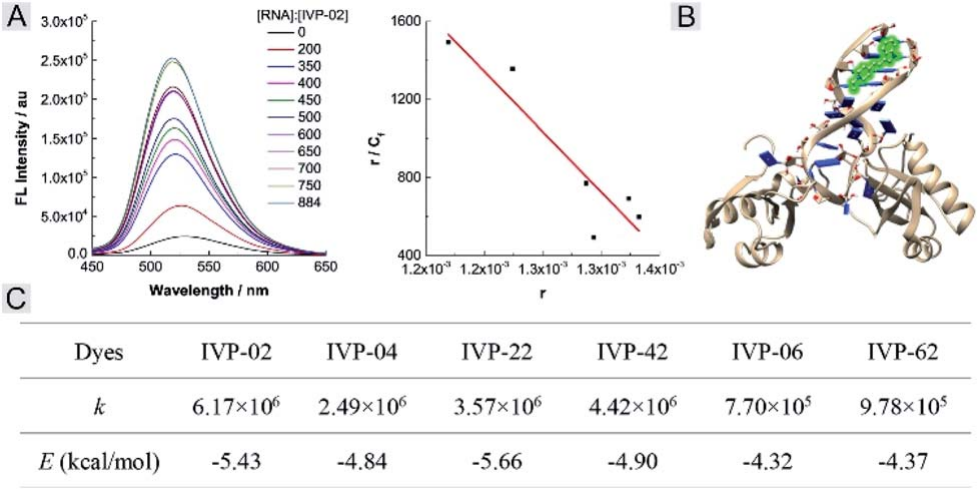
(A) Fluorescence titration (left) of **IVP-02** with RNA and the fitted curve (right) according to the Scatchard equation. (B) The binding mode of **IVP-02** to RNA. (C) Binding constant (*k*) and binding energy (*E*) of IVP molecules to RNA.

We further studied the reason that **IVP-06** and **62** cannot stain the nucleolus in cancer cells. On one hand, when IVP molecules are located in the mitochondria, in addition to the electrostatic interaction between the cation and negatively charged inner membrane of mitochondria, hydrophobic interaction between the alkyl chain and phospholipids also existed. When Δ*Ψ*_m_ decreased, although the electrostatic interaction weakened, the hydrophobic interaction still remained. **IVP-06** and **62** have longer alkyl chains than the other four IVP molecules, so the hydrophobic interaction between **IVP-06** and **62** and phospholipids is stronger than that of the other four IVP molecules. Therefore, **IVP-06** and **62** are more inclined to stay in the mitochondria, while **IVP-02**, **04**, **22**, and **42** more easily escape from the mitochondria. On the other hand, the affinity of IVP molecules to RNA should also be considered as the nucleolus is rich in RNA. Thus RNA titration experiments were first performed. In Fig. 9A and S24,† with the increase of RNA concentration, the fluorescence intensity of all the molecules increases. Based on the Scatchard equation,^54^ the binding constant (*k*) of these molecules to RNA was calculated and is summarized in Fig. 9C. It could be seen that the binding constant of **IVP-06** and **62** is lower than that of **IVP-02**, **04**, **22**, and **42**. Moreover, molecular docking calculations based on the structure of IVP molecules and RNA have also been performed. As shown in Fig. 9B and S24,† IVP molecules were bound to the minor grooves of RNA, and the binding energy (*E*) was calculated and is summarized in Fig. 9C. The calculated binding energy of **IVP-02**, **04**, **22**, and **42** is also higher than that of **IVP-06** and **62**, indicating that **IVP-02**, **04**, **22**, and **42** have stronger affinity to RNA than **IVP-06**, **62**.

### Two-photon imaging and cytotoxicity

**Fig. 10:**
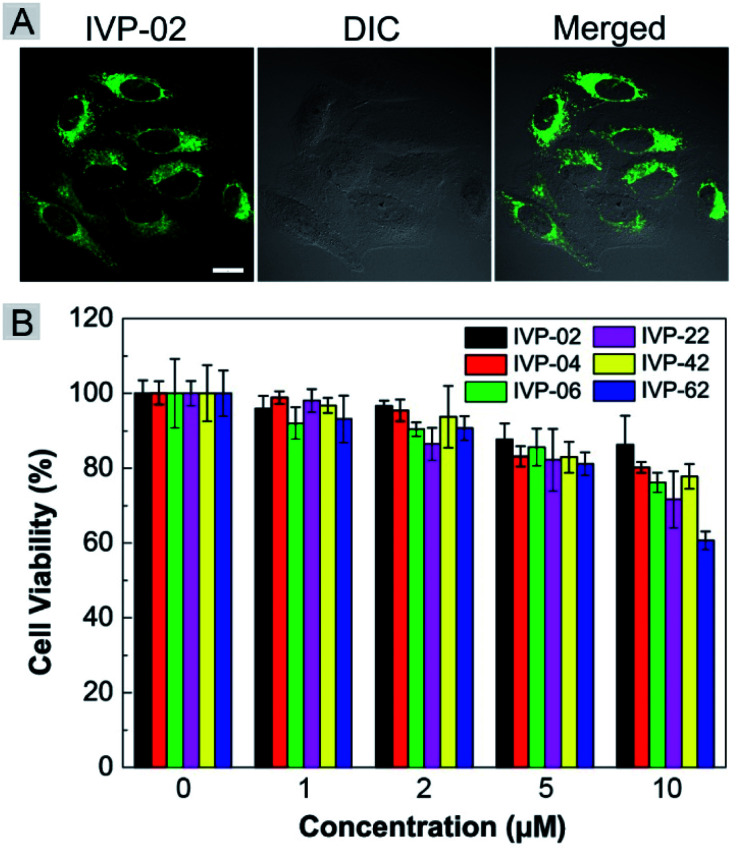
(A) Two-photon microscopy images of live HeLa cells stained with 2 μM **IVP-02** for 30 min. *λ*_ex_ = 800 nm, *λ*_em_ = 495–540 nm. Scale bar = 20 μm. (B) Viability of A549 cells after incubation with **IVP-02**, **04**, **06**, **22**, **42**, and **62** at different concentrations for 24 h.

Given the high two-photon absorption cross sectional values of **IVP-02** excited by 800 nm, we performed *in vitro* two-photon imaging of **IVP-02** in live HeLa cells. As displayed in Fig. 10A, bright two-photon fluorescence with high fidelity from filamentous structures of mitochondria in the cytoplasm could be clearly collected, demonstrating that **IVP-02** has great potential in two-photon imaging.

The potential long-term cytotoxicity of bioprobes should be carefully considered for imaging in live cells. Thus we studied the cytotoxicity of these IVP molecules in live A549 cells by the standard MTT assay. In Fig. 10B, it's clearly seen that the viability of A549 cells was higher than 80% after incubation with IVP molecules at a concentration less than 5 μM for 24 h, exhibiting very low cytotoxicity. When the incubation concentration was 10 μM, the cell viability was between 60% and 80%, showing certain cytotoxicity. Therefore, all cell imaging experiments in this work were conducted at a low concentration of 2 μM, which is reasonable and acceptable.

## Conclusions

To summarize, we have successfully synthesized a series of IVP molecules bearing different length alkyl chains. We mainly focus on two aspects. One is cancer cell discrimination. **IVP-02**, **22**, **42**, and **62** can distinguish cancer cells from normal cells because the membrane permeability and mitochondrial membrane potential of cancer cells is higher than those of normal cells. Compared with **IVP-02**, **22**, **42**, and **62**, more **IVP-04** and **06** molecules enter normal cells. The obvious difference between **IVP-02**, **22**, **42**, and **62**, and **IVP-04** and **06** is the length of the alkyl chain on the pyridinium salt side. The length of the alkyl chain on the pyridinium salt side determines the ionic radius. The longer the alkyl chain, the larger the ionic radius and the more easily it passes into the cell. **IVP-04** and **06** can stain cancer and normal cells simultaneously. The brightness in cancer cells is higher than that in normal cells because the mitochondrial membrane potential of cancer cells is higher than that of normal cells. The other aspect is cellular viability monitoring. **IVP-02**, **04**, **22**, and **42** can monitor the viability of cancer cells through mitochondria–nucleolus migration, while **IVP-06** and **62** cannot due to the stronger hydrophobic interaction between **IVP-06** and **62** and phospholipids and lower affinity to RNA. Finally, **IVP-02** has good two-photon properties and low cytotoxicity. These IVP molecules show excellent performance in cancer cell detection and cancer cell metastasis monitoring, and they show great potential in evaluating the activity and efficacy of drugs for cancer therapy. This work provides a theoretical and experimental basis for the design of other fluorescent probes to realize cancer cell discrimination and dynamic viability monitoring.

## Conflicts of interest

There are no conflicts to declare.

## Supplementary Material

SC-011-D0SC01213K-s001

SC-011-D0SC01213K-s002

## References

[cit1] Mousa S. (2010). Nanotechnol., Sci. Appl..

[cit2] Hartmann L. C., Keeney G. L., Lingle W. L., Christianson T. J. H., Varghese B., Hillman D., Oberg A. L., Low P. S. (2007). Int. J. Cancer.

[cit3] Nakagawa H., Liyanarachchi S., Davuluri R. V., Auer H., Martin E. W., de la Chapelle A., Frankel W. L. (2004). Oncogene.

[cit4] Wang Y., Zhou K., Huang G., Hensley C., Huang X., Ma X., Zhao T., Sumer B. D., DeBerardinis R. J., Gao J. (2014). Nat. Mater..

[cit5] Wu X., Liu H., Liu J., Haley K. N., Treadway J. A., Larson J. P., Ge N., Peale F., Bruchez M. P. (2003). Nat. Biotechnol..

[cit6] Cross S. E., Jin Y.-S., Rao J., Gimzewski J. K. (2007). Nat. Nanotechnol..

[cit7] Zamay T., Zamay G., Kolovskaya O., Zukov R., Petrova M., Gargaun A., Berezovski M., Kichkailo A. (2017). Cancers.

[cit8] Peng R., Zheng X., Lyu Y., Xu L., Zhang X., Ke G., Liu Q., You C., Huan S., Tan W. (2018). J. Am. Chem. Soc..

[cit9] Kanwar J. R., Roy K., Kanwar R. K. (2011). Crit. Rev. Biochem. Mol. Biol..

[cit10] Cerchia L., de Franciscis V. (2010). Trends Biotechnol..

[cit11] Ramirez C. N., Antczak C., Djaballah H. (2010). Expert Opin. Drug Discovery.

[cit12] Kumar P., Nagarajan A., Uchil P. D. (2018). Cold Spring Harb. Protoc..

[cit13] Colom A., Derivery E., Soleimanpour S., Tomba C., Molin M. D., Sakai N., González-Gaitán M., Matile S., Roux A. (2018). Nat. Chem..

[cit14] Yang Z., Sharma A., Qi J., Peng X., Lee D. Y., Hu R., Lin D., Qu J., Kim J. S. (2016). Chem. Soc. Rev..

[cit15] Zhu H., Fan J., Du J., Peng X. (2016). Acc. Chem. Res..

[cit16] Bai H., Lu H., Fu X., Zhang E., Lv F., Liu L., Wang S. (2018). Biomacromolecules.

[cit17] Klymchenko A. S. (2017). Acc. Chem. Res..

[cit18] Gao P., Pan W., Li N., Tang B. (2019). Chem. Sci..

[cit19] Jia R., Tian W., Bai H., Zhang J., Wang S., Zhang J. (2019). Nat. Commun..

[cit20] Xu W., Zeng Z., Jiang J.-H., Chang Y.-T., Yuan L. (2016). Angew. Chem., Int. Ed..

[cit21] Tian M., Ma Y., Lin W. (2019). Acc. Chem. Res..

[cit22] Wang Y., Feng L., Wang S. (2019). Adv. Funct. Mater..

[cit23] Tian M., Sun J., Tang Y., Dong B., Lin W. (2018). Anal. Chem..

[cit24] Bratosin D., Mitrofan L., Palii C., Estaquier J., Montreuil J. (2005). Cytometry, Part A.

[cit25] Vermes I., Haanen C., Steffens-Nakken H., Reutellingsperger C. (1995). J. Immunol. Methods.

[cit26] Crowley L. C., Scott A. P., Marfell B. J., Boughaba J. A., Chojnowski G., Waterhouse N. J. (2016). Cold Spring Harb. Protoc..

[cit27] He Z., Liu P., Zhang S., Yan J., Wang M., Cai Z., Wang J., Dong Y. (2019). Angew. Chem., Int. Ed..

[cit28] Chen C., Song Z., Zheng X., He Z., Liu B., Huang X., Kong D., Ding D., Tang B. Z. (2017). Chem. Sci..

[cit29] Hu Q., Gao M., Feng G., Liu B. (2014). Angew. Chem., Int. Ed..

[cit30] Xia F., Wu J., Wu X., Hu Q., Dai J., Lou X. (2019). Acc. Chem. Res..

[cit31] Gu K., Qiu W., Guo Z., Yan C., Zhu S., Yao D., Shi P., Tian H., Zhu W.-H. (2019). Chem. Sci..

[cit32] Bai H., Chen H., Hu R., Li M., Lv F., Liu L., Wang S. (2016). ACS Appl. Mater. Interfaces.

[cit33] Mei J., Leung N. L. C., Kwok R. T. K., Lam J. W. Y., Tang B. Z. (2015). Chem. Rev..

[cit34] Zalba S., ten Hagen T. L. M. (2017). Cancer Treat. Rev..

[cit35] Modica-Napolitano J. S., Aprille J. R. (2001). Adv. Drug Delivery Rev..

[cit36] Ly J. D., Grubb D. R., Lawen A. (2003). Apoptosis.

[cit37] Pollack L. (2011). Annu. Rev. Biophys..

[cit38] Murphy M. P. (2008). Biochim. Biophys. Acta, Bioenerg..

[cit39] Zhang R., Niu G., Li X., Guo L., Zhang H., Yang R., Chen Y., Yu X., Tang B. Z. (2019). Chem. Sci..

[cit40] Xue P., Wang P., Chen P., Yao B., Gong P., Sun J., Zhang Z., Lu R. (2017). Chem. Sci..

[cit41] Tong H., Hong Y., Dong Y., Häußler M., Lam J. W. Y., Li Z., Guo Z., Guo Z., Tang B. Z. (2006). Chem. Commun..

[cit42] Wang L., Yang L., Cao D. (2015). Sens. Actuators, B.

[cit43] Lu H., Xu B., Dong Y., Chen F., Li Y., Li Z., He J., Li H., Tian W. (2010). Langmuir.

[cit44] Pawlicki M., Collins H. A., Denning R. G., Anderson H. L. (2009). Angew. Chem., Int. Ed..

[cit45] Gui C., Zhao E., Kwok R. T. K., Leung A. C. S., Lam J. W. Y., Jiang M., Deng H., Cai Y., Zhang W., Su H., Tang B. Z. (2017). Chem. Sci..

[cit46] Jiang N., Fan J., Xu F., Peng X., Mu H., Wang J., Xiong X. (2015). Angew. Chem., Int. Ed..

[cit47] Guo L., Li C., Shang H., Zhang R., Li X., Lu Q., Cheng X., Liu Z., Sun J. Z., Yu X. (2020). Chem. Sci..

[cit48] Ross M. F., Kelso G. F., Blaikie F. H., James A. M., Cochemé H. M., Filipovska A., Da Ros T., Hurd T. R., Smith R. A. J., Murphy M. P. (2005). Biochemistry.

[cit49] Nunnari J., Suomalainen A. (2012). Cell.

[cit50] Balaban R. S., Nemoto S., Finkel T. (2005). Cell.

[cit51] Li X., Tian M., Zhang G., Zhang R., Feng R., Guo L., Yu X., Zhao N., He X. (2017). Anal. Chem..

[cit52] Lim M. L. R., Minamikawa T., Nagley P. (2001). FEBS Lett..

[cit53] Zhang X., Lee M. D., Wilson C., McCarron J. G. (2019). Cell Calcium.

[cit54] Tian M., Sun J., Dong B., Lin W. (2018). Angew. Chem., Int. Ed..

