# Supplementary material for: Cancer cell discrimination and dynamic viability monitoring through wash-free bioimaging using AIEgens
Source: Chem Sci. 2020 Apr 30;11(29):7676–84. doi: 10.1039/d0sc01213k (PMC8159538; doi:10.1039/d0sc01213k)
Supplement: SC-011-D0SC01213K-s001 [file SC-011-D0SC01213K-s001.pdf]

Electronic Supplementary Material (ESI) for Chemical Science.
This journal is © The Royal Society of Chemistry 2020

Cancer cell discrimination and dynamic viability monitoring through wash-free bioimaging by AIEgens

Ruoyao Zhang,[a,b] *Guangle Niu,*[a,b,c] *Qing Lu,*[c] *Xiaolin Huang,*[a,b] *Joe H. C. Chau,*[a] *Ryan T. K. Kwok,*[a,b] *Xiaoqiang Yu,*[c] *Min-Hui Li,*[d,*] *Jacky W. Y. Lam,*[a,b*] *and Ben Zhong Tang*[a,b,e*]

[a] Department of Chemistry, Hong Kong Branch of Chinese National Engineering Research Center for Tissue Restoration and Reconstruction, Institute for Advanced Study, and Department of Chemical and Biological Engineering, The Hong Kong University of Science and Technology, Clear Water Bay, Kowloon, Hong Kong 999077, China. E-mail: chjacky@ust.hk; tangbenz@ust.hk

[b] HKUST-Shenzhen Research Institute, No. 9 Yuexing 1st RD, South Area, Hi-tech Park, Nanshan, Shenzhen 518057, China

[c] Center of Bio and Micro/Nano Functional Materials, State Key Laboratory of Crystal Materials, Shandong University, Jinan 250100, China

[d] Chimie ParisTech PSL University Paris, CNRS, Institut de Recherche de Chimie Paris 75005 Paris (France). E-mail: min-hui.li@chimieparistech.psl.eu

[e] Center for Aggregation-Induced Emission, SCUT-HKUST Joint Research Institute, State Key Laboratory of Luminescent Materials and Devices, South China University of Technology, Guangzhou 510640, China

*Corresponding author:

Jacky W. Y. Lam; E-mail: chjacky@ust.hk

Min-Hui Li; E-mail: min-hui.li@chimieparistech.psl.eu

Ben Zhong Tang; E-mail: tangbenz@ust.hk

1. Materials and Methods

1.1 Apparatus and general methods

All the chemicals were purchased and used as received without further purification unless otherwise specified. MitoTracker Deep Red FM (MTDR) was purchased from life technology. Ribonucleic acid from torula yeast and carbonyl cyanide *m*-chlorophenyl hydrazone (CCCP) were purchased form Sigma. The UV-Vis absorption spectra of dilute solutions were recorded on a Milton Roy Spectronic 3000 Array spectrameter using a quartz cuvette with 1 cm path length. Fluorescence spectra were obtained on a Perkin-Elmer LS 55 spectrofluometer equipped with a temperature controller. All the fluorescence quantum yields were measured using an integrating sphere.

1.2 Measurement of two-photon absorption cross section

Two-photon absorption cross-section (δ) was measured using the two-photon induced fluorescence method, and thus the δ can be calculated by means of Eq. (1)[1]:

$$\delta_s = \delta_r \frac{\Phi_r c_r n_r F_s}{\Phi_s c_s n_s F_r} \qquad (1)$$

F is TPEF integral intensity. Φ is the fluorescence quantum yield. δ_r is the two-photon absorption cross-section of fluorescein in sodium hydroxide aqueous solution (pH = 13.0)[1].

1.3 Cell culture and staining

Cell culture: human lung adenocarcinoma cells (A549), Human cervical cancer cells (HeLa), human lung fibroblasts (HLF), and African green monkey kidney fibroblasts (COS7) were grown in Dulbecco's Modified Eagle Medium supplemented with 10% fetal bovine serum (FBS) and 1% penicillin and streptomycin in a 5% CO_2 incubator at 37 ^{o}C.

Cell staining experiments: IVP-02, 04, 06, 22, 42, and 62 were dissolved in DMSO at a stock concentration of 1 mM, respectively. A549, HeLa, COS7, and HLF cells were placed on glass coverslips and allowed to adhere for 48 h. A549, HeLa, COS7, and HLF

cells were incubated with these IVP probes (2 μM) in DMEM at 37 ^{o}C for 30 min. Co-staining experiments: MTDR was dissolved in DMSO at a stock concentration of 0.1 mM. To confirm the location of these IVP molecules in mitochondria in cancer cells, HeLa and A549 cells were firstly incubated with 0.2 μM MTDR for 15 min, then stained with 2 μM IVP molecules for 30 min, respectively.

1.4 Fluorescence imaging

The confocal fluorescent images were obtained with Zeiss LSM 800 confocal laser scanning microscope. The co-localization coefficient and mean fluorescence intensity of the images were determined by the software with Zeiss LSM 800 confocal microscope. Two-photon microscopy images were obtained with Olympus FV 1200 laser scanning microscope.

1.5 RNA titration

Stock solutions of IVP molecules were initially prepared in the solvent of DMSO with the concentration of 5 mM. Stock solution of RNA was prepared in the concentration of 1 mg/mL in Tris buffer solutions (pH = 7.2). To calculate the molar concentration of the stock solution, RNA solution was diluted to 0.01 ‰ and the absorption spectra were measured. The molar concentration of RNA stock solutions was then calculated with the following Eq. (2)[2]:

$$C_{RNA} = \frac{A_{260}}{6600} \times 100000\, mol/L \tag{2}$$

Where C_{RNA} is the concentration of the stock solution, A_{260} is the absorbance at 260 nm.

In RNA titration experiments, suitable amount of the stock solutions of IVP molecules and RNA was added into Tris buffer solutions (pH = 7.2) to obtain different concentrations. Then the absorption spectra were acquired, and the fluorescence spectra were also measured with the spectrofluorimeter with the excitation wavelength of 400 nm.

1.6 Molecular simulation

The chemical structures of the fluorescent probes were initially optimized with

Gaussian 09 software package. A series of calculations were performed sequentially with the basic set of pm3, b3lyp/3-21g, and b3lyp/6-31g. The final chemical structures were used for the following docking calculations.

For molecular docking calculations, the optimized structures of the three probes were used. The structure of RNA was downloaded from the RCSB Protein Data Bank. AutoDock 4.2 software was used for the docking calculations. The number of GA runs was set as 50, the maximum number of evals was set as medium, and other parameters were set as the default values. After docking calculation, the most stable conformation was used for analysis.

1.7 MTT assay

A549 cells growing in log phase were seeded into 96-well plates (ca. 1×10^4 cells/well) and allowed to adhere for 24 h. IVP molecules dissolved in DMEM at concentrations of 1 μM, 2 μM, 5 μM, and 10 μM, respectively, were added into the wells as the treatment group (200 μL/well), and DMEM without dye was added into the wells as the negative control group. The cells were incubated for 24 h at 37 °C under 5% CO_2. Then MTT (5 mg/mL in DMEM, 20 μL) was added into each well. After 4 h incubation at 37 °C, 100 μL DMSO was added to dissolve the purple crystals. After 20 min incubation, the optical density readings at 570 nm were taken using a plate reader. Cytotoxic experiment was repeated for three times.

2. Crystallographic data

Table S1 Crystal data and structure refinement for IVP-02.

```
Bond precision:   C-C = 0.0049 A          Wavelength=1.54178

Cell:             a=6.8004(9)     b=11.9948(15)      c=21.500(3)
                  alpha=90        beta=95.373(4)     gamma=90
Temperature:      150 K

                 Calculated                 Reported
Volume           1746.0(4)                  1746.0(4)
Space group      P 21/n                     P 21/n
Hall group       -P 2yn                     -P 2yn
Moiety formula   C17 H17 N2, F6 P           ?
Sum formula      C17 H17 F6 N2 P            C17 H17 F6 N2 P
Mr               394.30                     394.29
Dx,g cm-3        1.500                      1.500
Z                4                          4
Mu (mm-1)        2.005                      2.005
F000             808.0                      808.0
F000'            812.29
h,k,lmax         8,15,26                    8,14,26
Nref             3585                       3487
Tmin,Tmax        0.486,0.548                0.501,0.585
Tmin'            0.427

Correction method= # Reported T Limits: Tmin=0.501 Tmax=0.585
AbsCorr = MULTI-SCAN

Data completeness= 0.973          Theta(max)= 74.619

R(reflections)= 0.0851( 3291)     wR2(reflections)= 0.2315( 3487)

S = 1.048                  Npar= 236
```

3. Photophysical properties

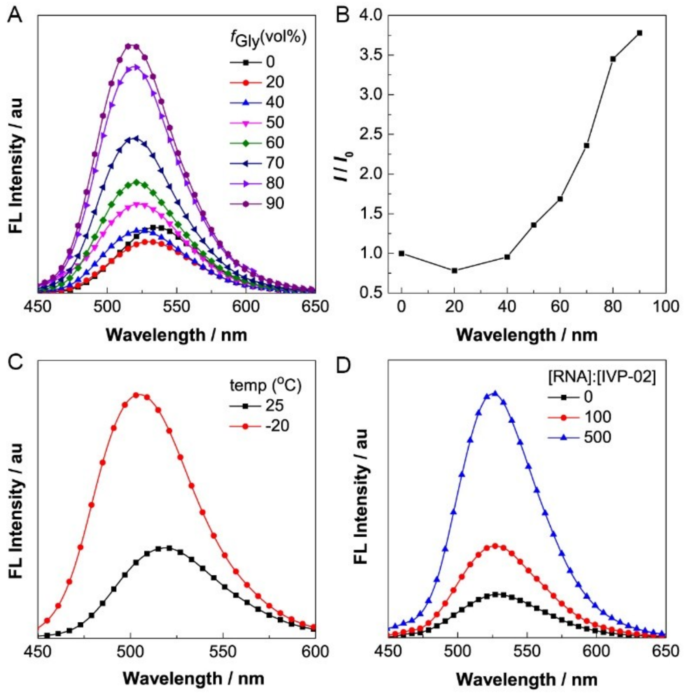

Fig. S1 (A) FL spectra of IVP-02 in MeOH and MeOH/glycerol mixtures with different glycerol fractions (f_{Gly}). (B) Changes in the FL peak intensities (I) of the solutions of IVP-02 with the glycerol contents in the MeOH/glycerol mixtures. I_0 is the intensity in pure MeOH. (C) FL spectra of IVP-02 in MeOH/glycerol mixture with 90% glycerol at 25 and -20 ℃. Concentration: 10 μM. (D) FL spectra of IVP-02 (5 μM) in PBS with different RNA concentrations.

Table S2 Two-photon absorption cross section (δ) of IVP-02 in DMSO.

λ_{ex} / nm	780	800	820	840	860	880	900
δ / GM	106	287	173	194	271	227	78

4. Bioimaging

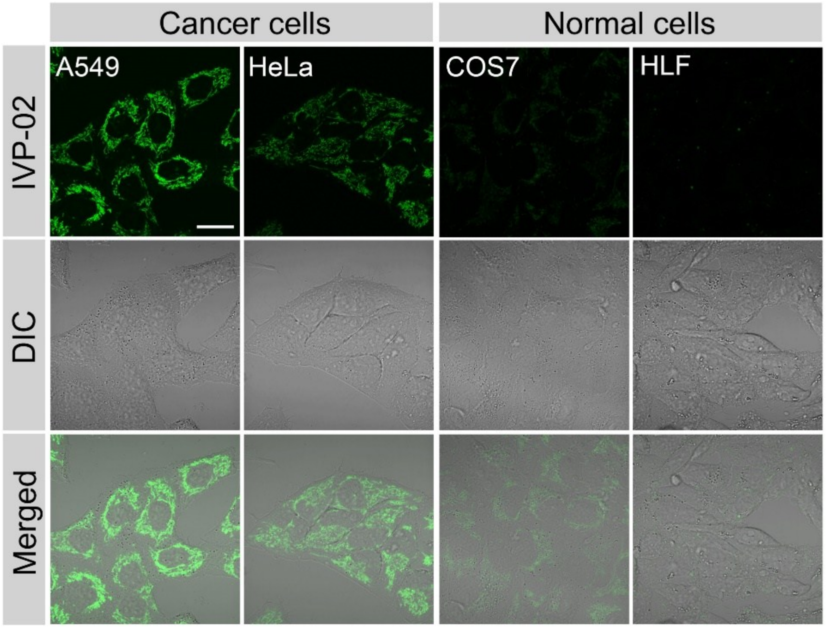

Fig. S2 CLSM images of live cancer cells (A549 and HeLa) and normal cells (COS7 and HLF) stained with 2 μM IVP-02 for 30 min, respectively. λ_{ex}= 488 nm, λ_{em}= 500-650 nm. Scale bar = 20 μm.

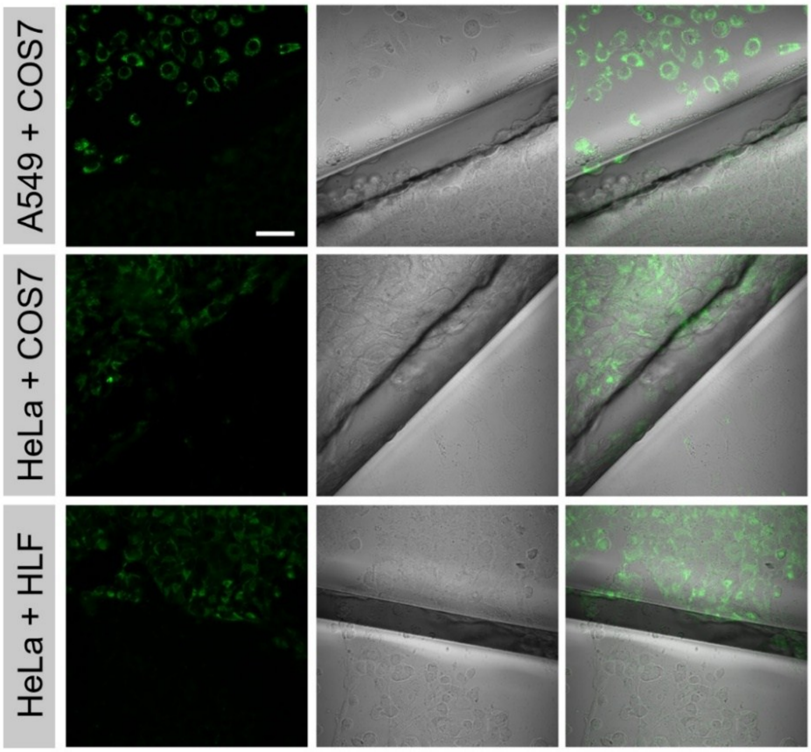

Fig. S3 CLSM images of live cancer cells (A549 and HeLa) co-cultured with normal cells (COS7 and HLF) seeded on different cover glasses stained with 2 μM IVP-02 for 30 min, respectively. λ_{ex}=

488 nm, λ_{em}= 500-650 nm. Scale bar = 50 μm.

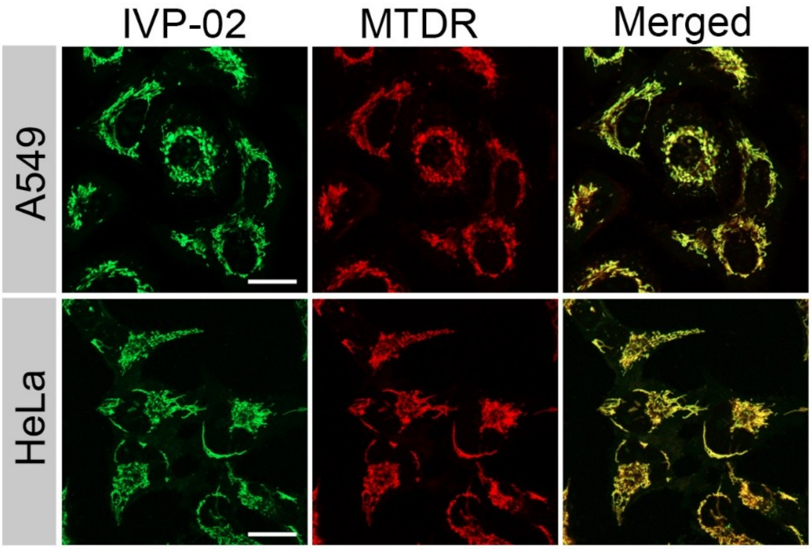

Fig. S4 CLSM images of live HeLa and A549 cells stained with 2 μM IVP-02 and 0.2 μM MTDR, respectively. IVP-02: λ_{ex}= 488 nm, λ_{em}= 500-650 nm; MTDR: λ_{ex}= 640 nm, λ_{em}= 650-700 nm. Scale bar = 20 μm.

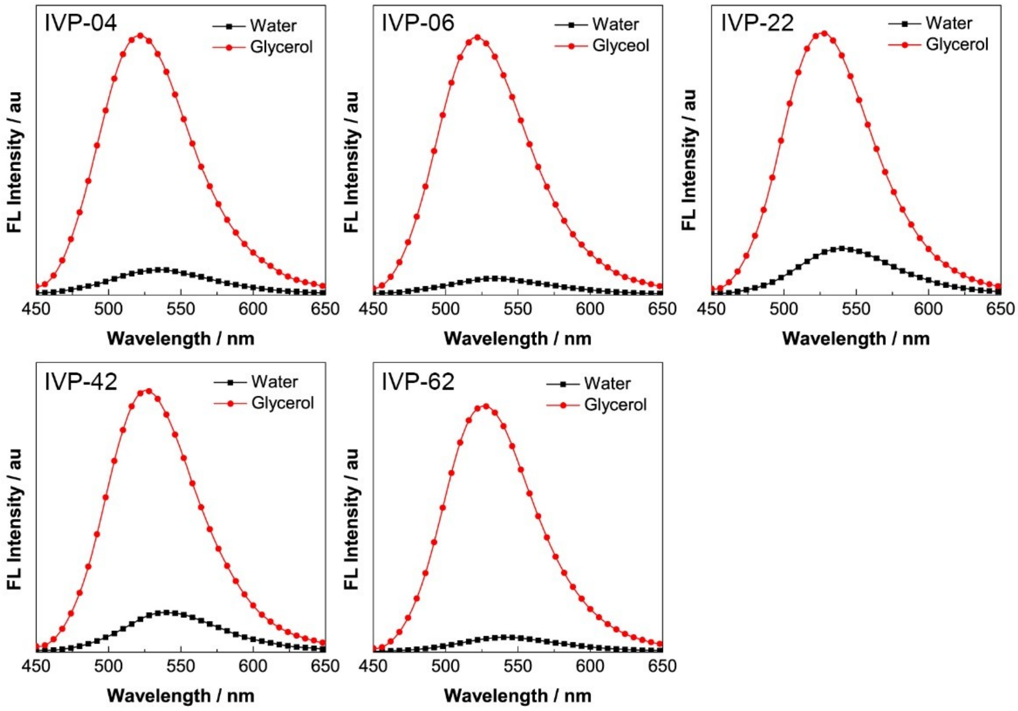

Fig. S5 FL spectra of IVP-04, IVP-06, IVP-22, IVP-42, and IVP-62 in water and glycerol, respectively. Concentration: 10 μM.

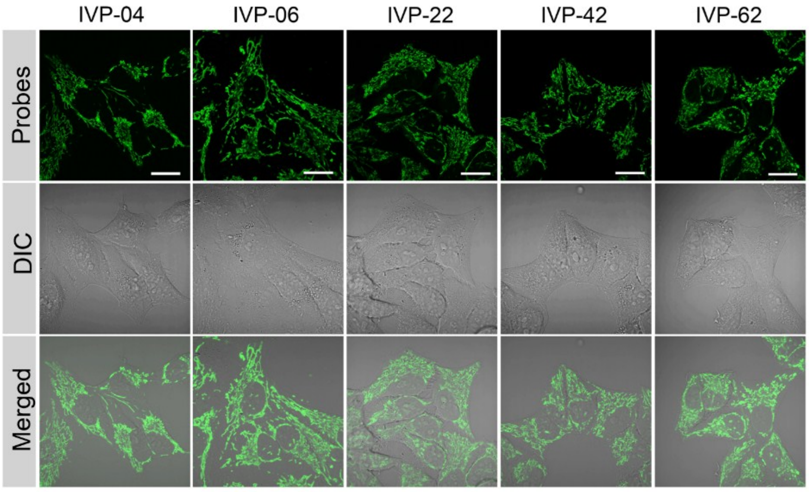

Fig. S6 CLSM images of live HeLa cells with stained 2 μM IVP-04, 06, 22, 42, and 62 for 30 min, respectively. λ_{ex}= 488 nm, λ_{em}= 500-650 nm. Scale bar = 20 μm.

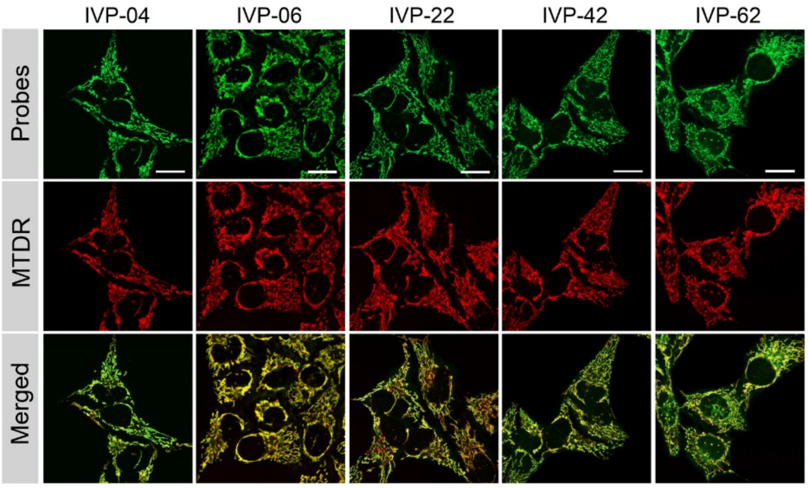

Fig. S7 CLSM images of HeLa cells stained with 2 μM IVP-04, 06, 22, 42, 62, and 0.2 μM MTDR, respectively. IVP-04, 06, 22, 42, and 62: λ_{ex}= 488 nm, λ_{em}= 500-650 nm; MTDR: λ_{ex}= 640 nm, λ_{em}= 650-700 nm. Scale bar = 20 μm.

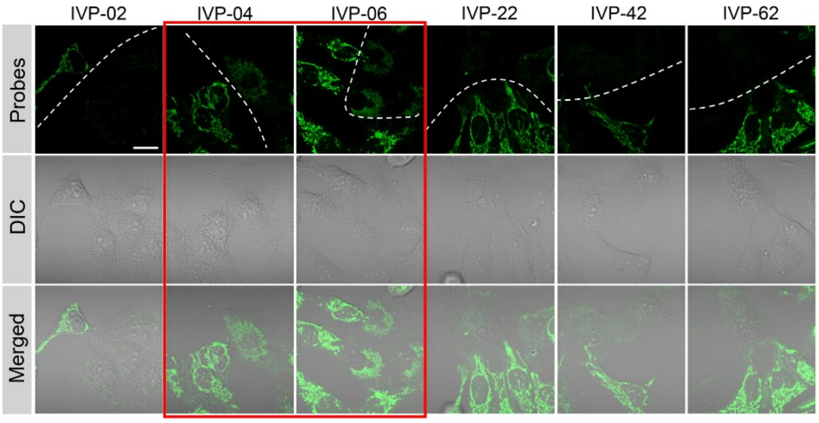

Fig. S8 CLSM images of live cancer cells (HeLa) co-cultured with normal cells (COS7), stained

with 2 μM IVP-02, 04, 06, 22, 42, and 62 for 30 min, respectively. λ_{ex}= 488 nm, λ_{em}= 500-650 nm. Scale bar = 20 μm.

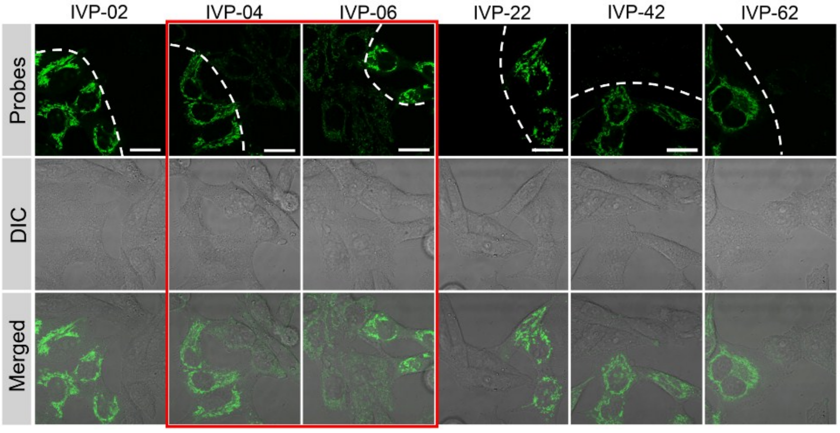

Fig. S9 CLSM images of live cancer cells (A549) co-cultured with normal cells (HLF), stained with 2 μM IVP-02, 04, 06, 22, 42, and 62 for 30 min, respectively. λ_{ex}= 488 nm, λ_{em}= 500-650 nm. Scale bar = 20 μm.

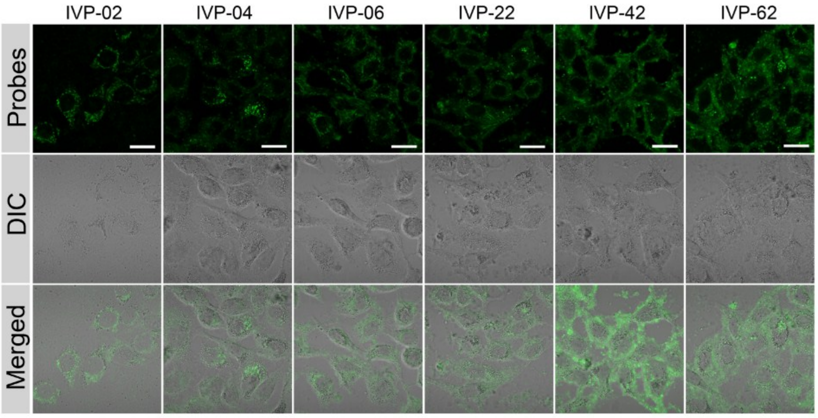

Fig. S10 CLSM images of live A549 cells 2 μM IVP-02, 04, 06, 22, 42, and 62 at 4 ℃ for 20 min, respectively. λ_{ex}= 488 nm, λ_{em}= 500-650 nm. Scale bar = 20 μm.

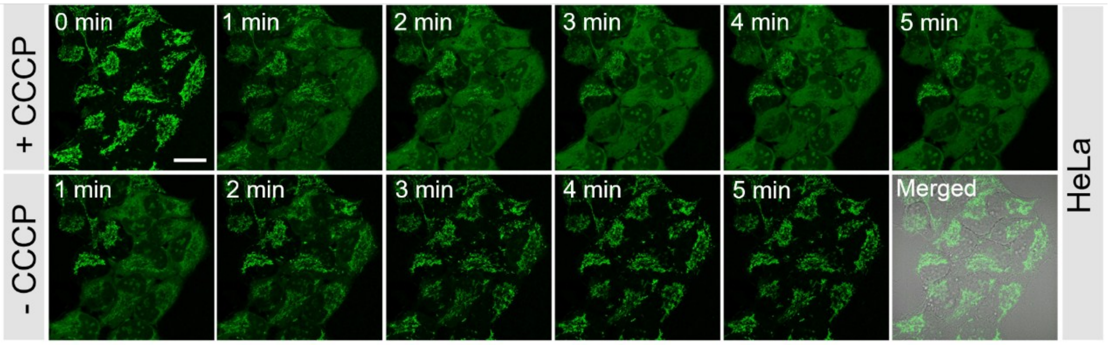

Fig. S11 Live HeLa cells were pre-stained with 2 μM IVP-02 for 30 min, and treated with 20 μM CCCP for 5 min. Then CCCP was removed away and fresh culture medium was added for another

5 min. λ_{ex}= 488 nm, λ_{em}= 500-650 nm. Scale bar = 20 μm.

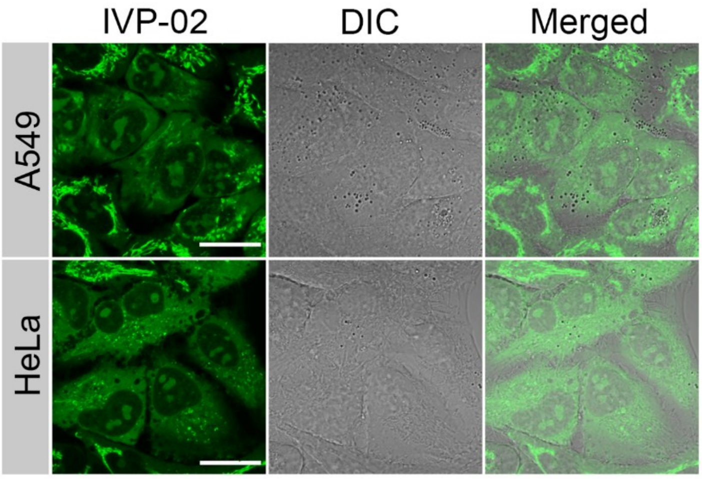

Fig. S12 Live A549 and HeLa cells were pre-stained with 2 μM IVP-02 for 30 min, and treated with 10 mM H_2O_2 for 10 min. λ_{ex}= 488 nm, λ_{em}= 500-650 nm. Scale bar = 20 μm.

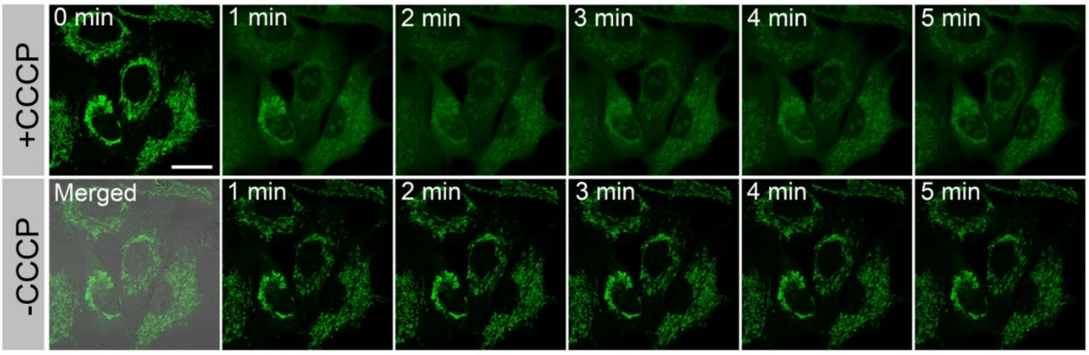

Fig. S13 Live A549 cells were pre-stained with 2 μM IVP-04 for 30 min, and treated with 20 μM CCCP for 5 min, Then CCCP was removed away and fresh culture medium was added for another 5 min. λ_{ex}= 488 nm, λ_{em}= 500-650 nm. Scale bar = 20 μm.

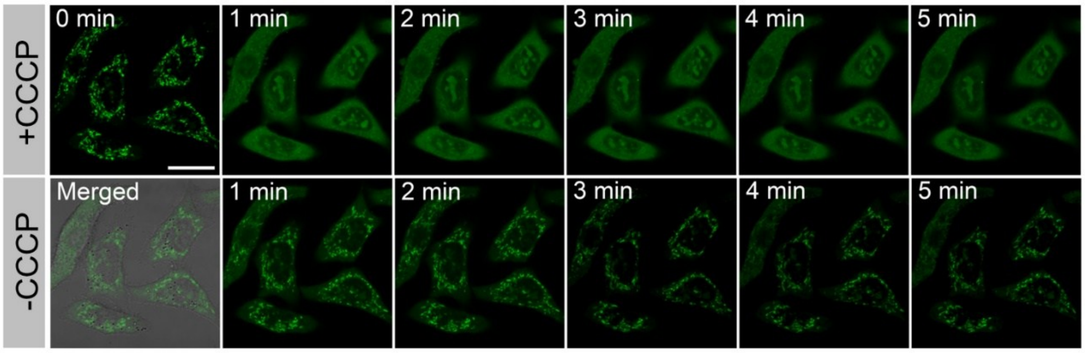

Fig. S14 Live A549 cells were pre-stained with 2 μM IVP-22 for 30 min, and treated with 20 μM CCCP for 5 min, Then CCCP was removed away and fresh culture medium was added for another

5 min. λ_{ex}= 488 nm, λ_{em}= 500-650 nm. Scale bar = 20 μm.

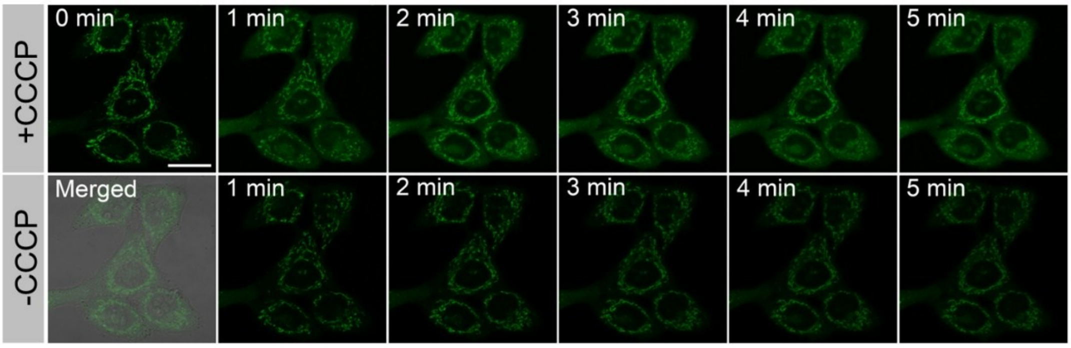

Fig. S15 Live A549 cells were pre-stained with 2 μM IVP-42 for 30 min, and treated with 20 μM CCCP for 5 min, Then CCCP was removed away and fresh culture medium was added for another 5 min. λ_{ex}= 488 nm, λ_{em}= 500-650 nm. Scale bar = 20 μm.

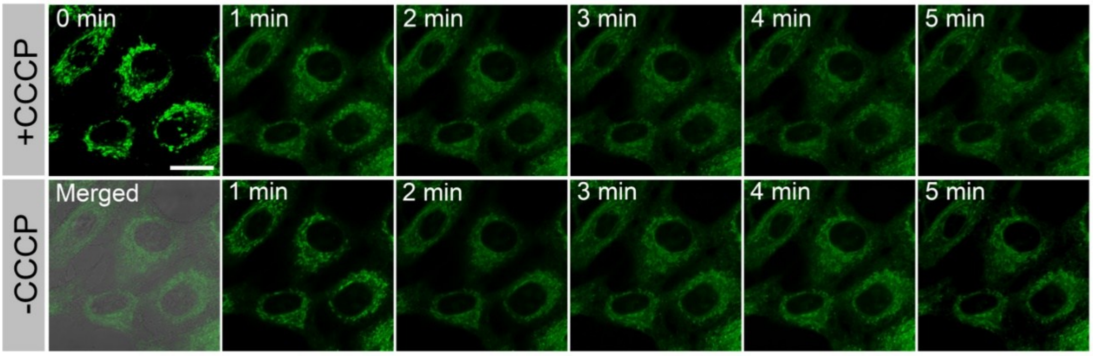

Fig. S16 Live A549 cells were pre-stained with 2 μM IVP-06 for 30 min, and treated with 20 μM CCCP for 5 min, Then CCCP was removed away and fresh culture medium was added for another 5 min. λ_{ex}= 488 nm, λ_{em}= 500-650 nm. Scale bar = 20 μm.

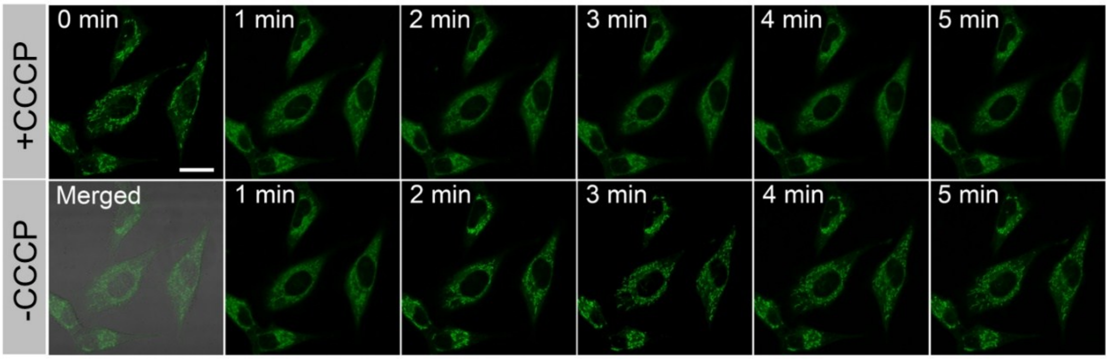

Fig. S17 Live A549 cells were pre-stained with 2 μM IVP-62 for 30 min, and treated with 20 μM CCCP for 5 min, Then CCCP was removed away and fresh culture medium was added for another 5 min. λ_{ex}= 488 nm, λ_{em}= 500-650 nm. Scale bar = 20 μm.

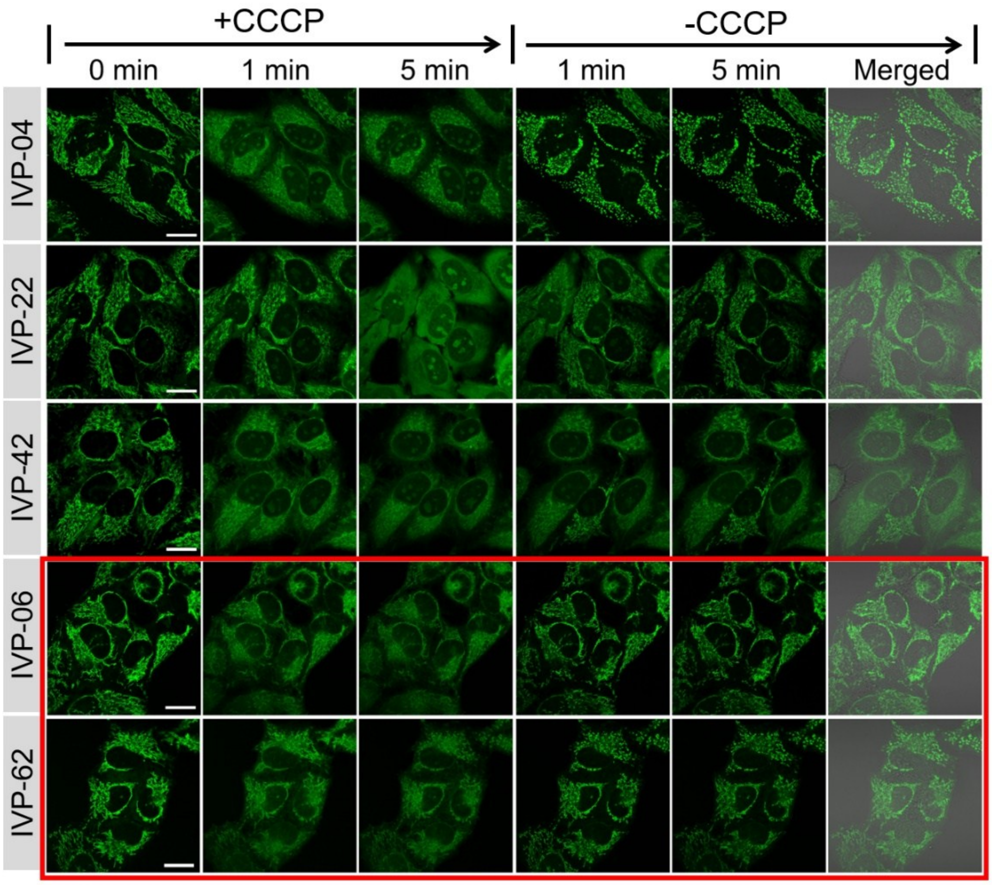

Fig. S18 Live HeLa cells were pre-stained with 2 μM IVP-04, 22, 42, 06, 62 for 30 min, and treated with 20 μM CCCP for 5 min. Then CCCP was removed away and fresh culture medium was added for another 5 min, respectively. λ_{ex}= 488 nm, λ_{em}= 500-650 nm. Scale bar = 20 μm.

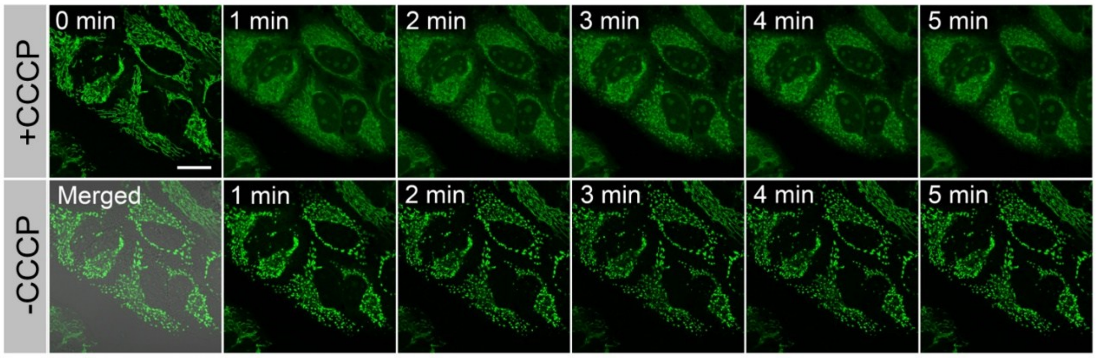

Fig. S19 Live HeLa cells were pre-stained with 2 μM IVP-04 for 30 min, and treated with 20 μM CCCP for 5 min, Then CCCP was removed away and fresh culture medium was added for another 5 min. λ_{ex}= 488 nm, λ_{em}= 500-650 nm. Scale bar = 20 μm.

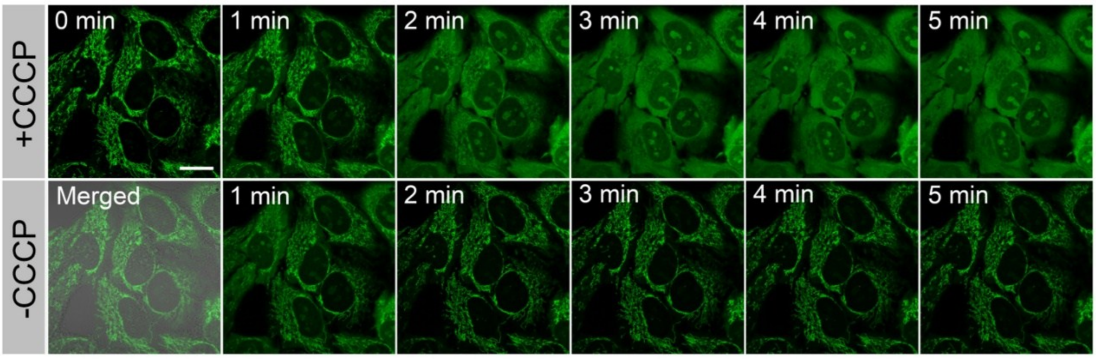

Fig. S20 Live HeLa cells were pre-stained with 2 μM IVP-22 for 30 min, and treated with 20 μM CCCP for 5 min, Then CCCP was removed away and fresh culture medium was added for another 5 min. λ_{ex}= 488 nm, λ_{em}= 500-650 nm. Scale bar = 20 μm.

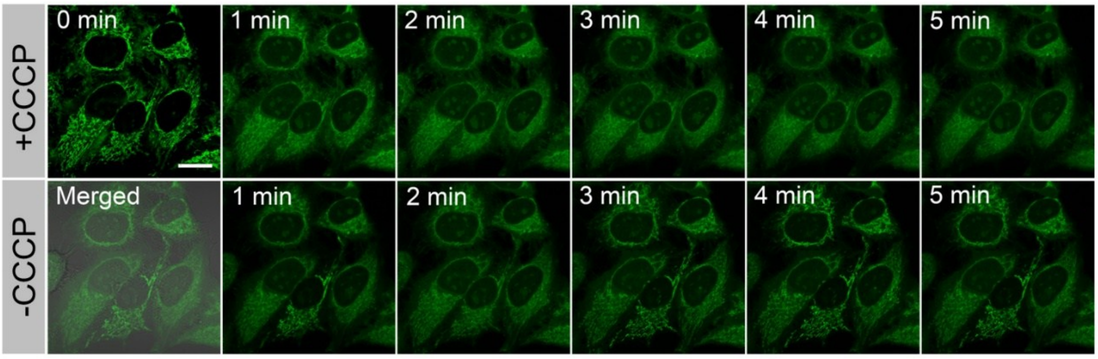

Fig. S21 Live HeLa cells were pre-stained with 2 μM IVP-42 for 30 min, and treated with 20 μM CCCP for 5 min, Then CCCP was removed away and fresh culture medium was added for another 5 min. λ_{ex}= 488 nm, λ_{em}= 500-650 nm. Scale bar = 20 μm.

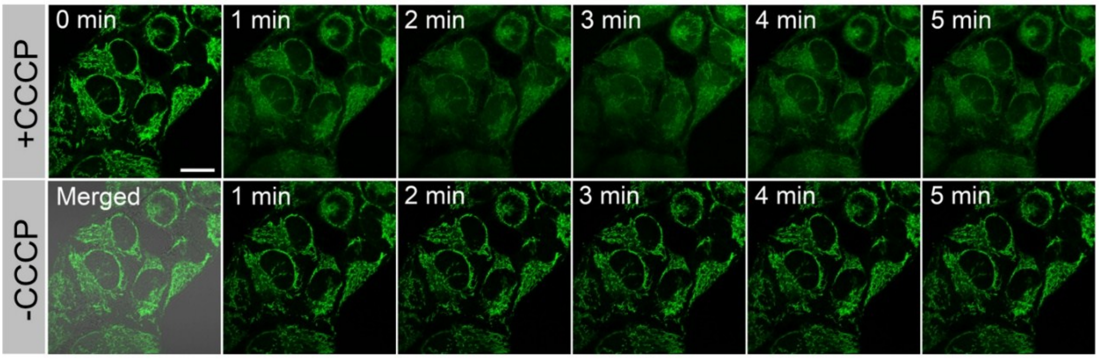

Fig. S22 Live HeLa cells were pre-stained with 2 μM IVP-06 for 30 min, and treated with 20 μM CCCP for 5 min, Then CCCP was removed away and fresh culture medium was added for another 5 min. λ_{ex}= 488 nm, λ_{em}= 500-650 nm. Scale bar = 20 μm.

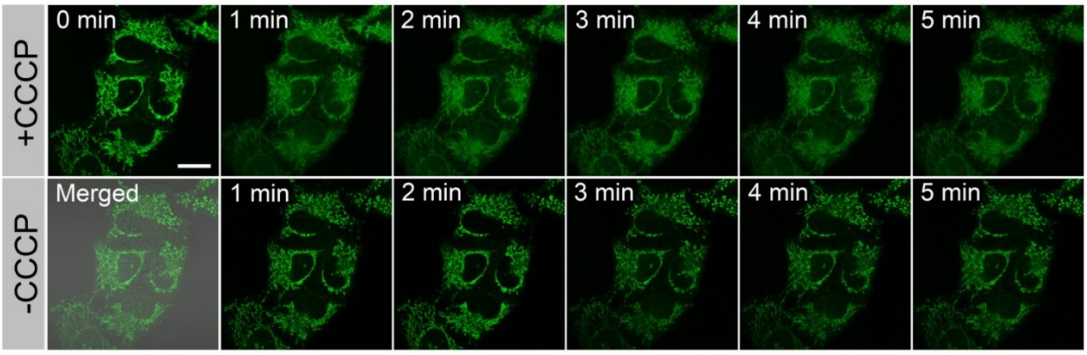

Fig. S23 Live HeLa cells were pre-stained with 2 μM IVP-62 for 30 min, and treated with 20 μM CCCP for 5 min, Then CCCP was removed away and fresh culture medium was added for another 5 min. λ_{ex}= 488 nm, λ_{em}= 500-650 nm. Scale bar = 20 μm.

5. RNA titration and calculations

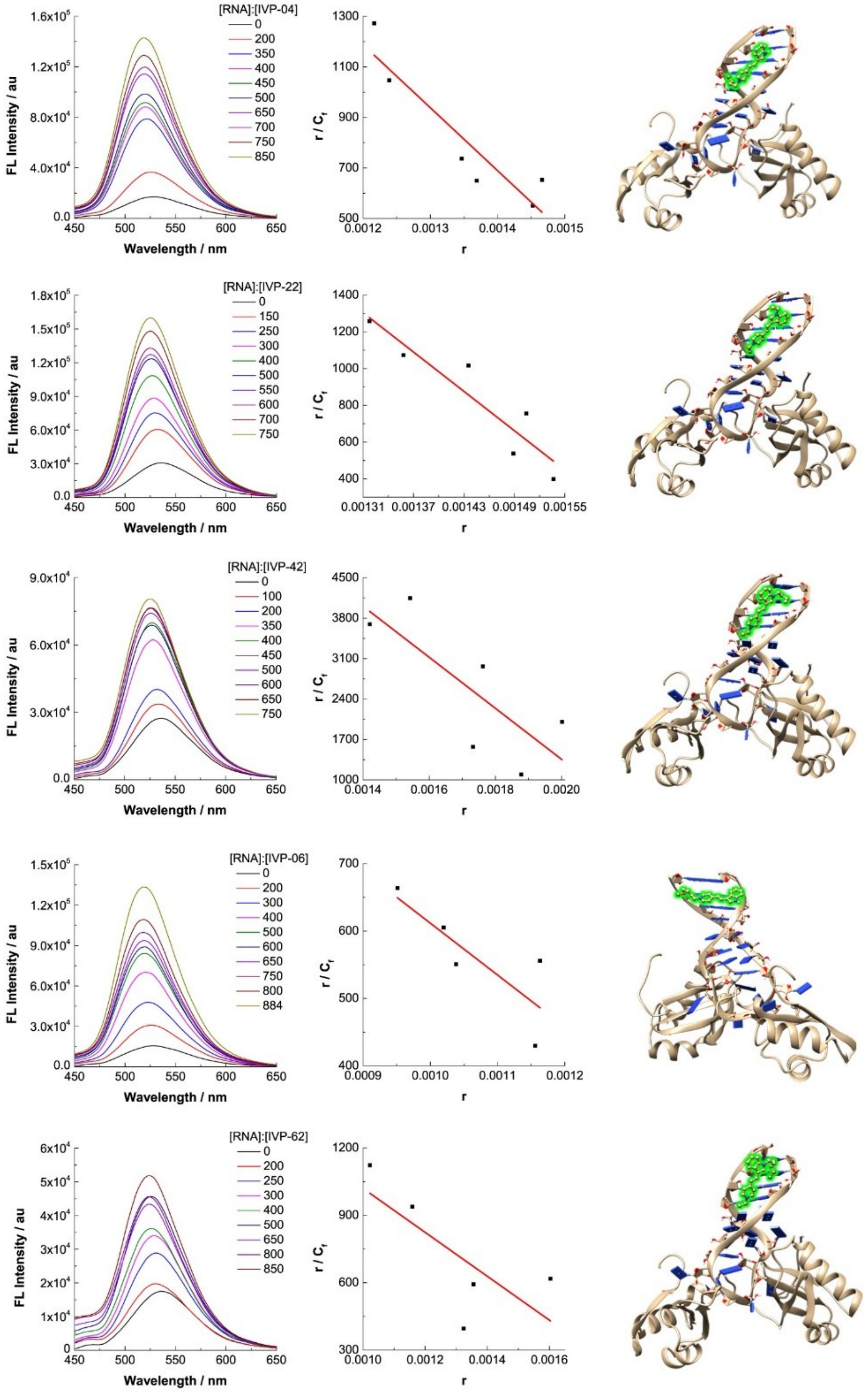

Fig. S24 Fluorescence titration (left) of IVP-04, 22, 42, 06, and 62 with RNA and the fitted curve (middle) according to Scatchard equation, and the binding mode (right) of them to RNA.

6. Synthetic details, NMR spectra and HRMS spectra

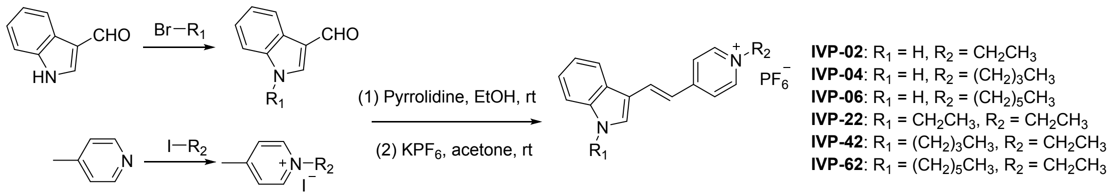

Scheme S1. Synthesis routes of IVP-02, 04, 06, 22, 42, and 62.

Since the synthesis routes and experimental procedures of the IVP molecules are very similar. Here we take IVP-02 as an example.

Compound 1-ethyl-4-methylpyridin-1-ium iodide was first synthesized. 4-methylpyridine (1mL, 10 mmol) was dissolved in ethanol (5 mL) and iodoethane (1 mL, 12 mmol) was added. The mixture was stirred at 78 °C overnight. Then the reaction mixture was poured into petroleum ether, and the light yellow solid was filtrated. After recrystallization, compound 1-ethyl-4-methylpyridin-1-ium iodide was obtained as a light yellow solid (2.24 g, 90%). Then IVPI-2 was synthesized. Pyrrolidine (200 μL) was added to the solution of 1*H*-indole-3-carbaldehyde (0.15 g, 1 mmol) and compound 1-ethyl-4-methylpyridin-1-ium iodide (0.25 g, 1 mmol) dissolved in methanol (5 mL). The mixture was stirred at room temperature for 5 h. Then the reaction mixture was poured into petroleum ether, and the orange solid was filtrated. After recrystallization, IVPI-12 was obtained as an orange solid (0.26 g, 65%). Finally, IVP-02 was synthesized. IVPI-2 (0.11 g, 0.3 mmol) was dissolved in acetone (10 mL), and a solution of KPF_6 (0.55 g, 3 mmol) in was added. The mixture was stirred at room temperature for 24 h. Acetone was removed under reduced pressure and the residue was purified by silica gel chromatography using CH_2Cl_2/MeOH mixture (8:1, v/v) as eluent to give IVP-02 as an orange red solid (66 mg, 56%). ^{1}H NMR (400 MHz, DMSO-d_6), δ (ppm): 11.92 (s, 1H), 8.77 (d, J = 6.60 Hz, 2H), 8.25 (d, J = 16.20 Hz, 1H), 8.15 (dd, J = 11.70 Hz, 6.80 Hz, 3H), 7.97 (s, 1H), 7.51 (dd, J = 6.90 Hz, 1.90 Hz, 1H), 7.34-7.19 (m, 3H), 4.44 (q, J = 7.30 Hz, 2H), 1.51 (t, J = 7.30 Hz, 3H). ^{13}C NMR (100 MHz, MeOD-d_4), δ (ppm): 155.14, 141.82, 137.43, 136.53, 131.42, 127.88, 124.43, 122.33, 121.28, 120.65, 119.31, 115.68, 113.49, 111.38, 54.41, 14.56. ^{19}F NMR (376 MHz, MeOD-d_4), δ (ppm): (-73.73, 3F), (-75.61, 3F). HRMS m/z: calcd for $C_{17}H_{17}N_2^+$

249.1386, found 249.1375 ([M]$^+$).

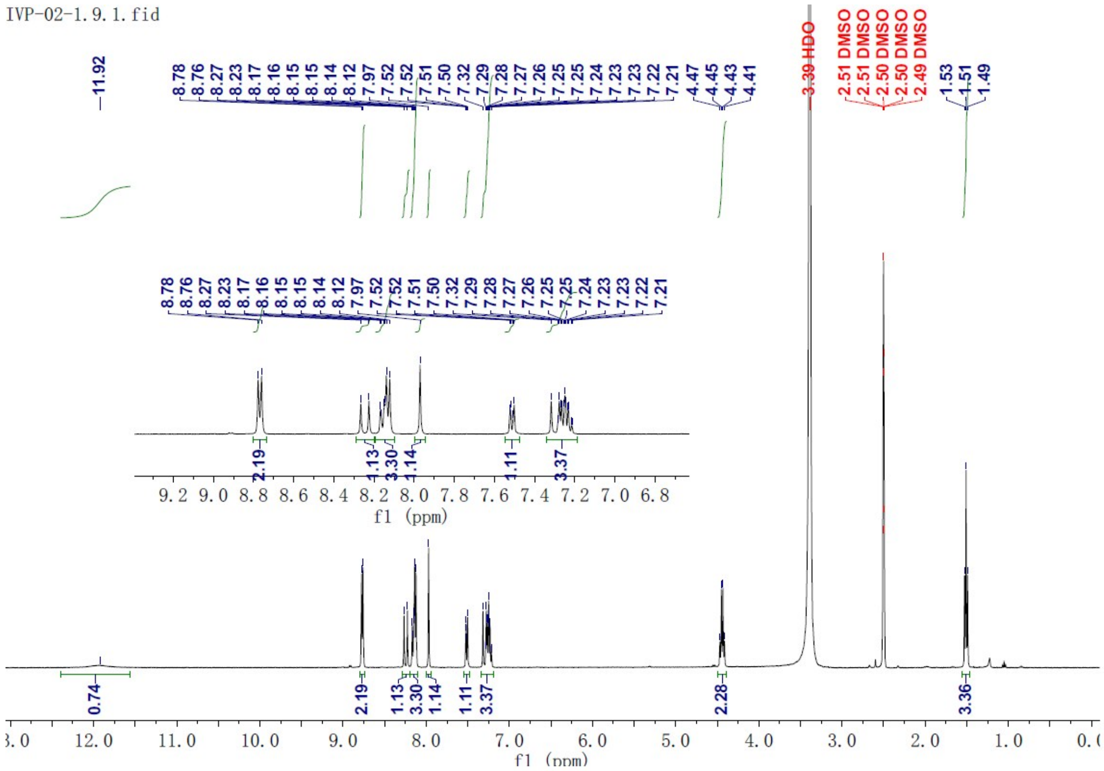

Fig. S25. ^{1}H NMR spectrum of IVP-02 in DMSO-d_6.

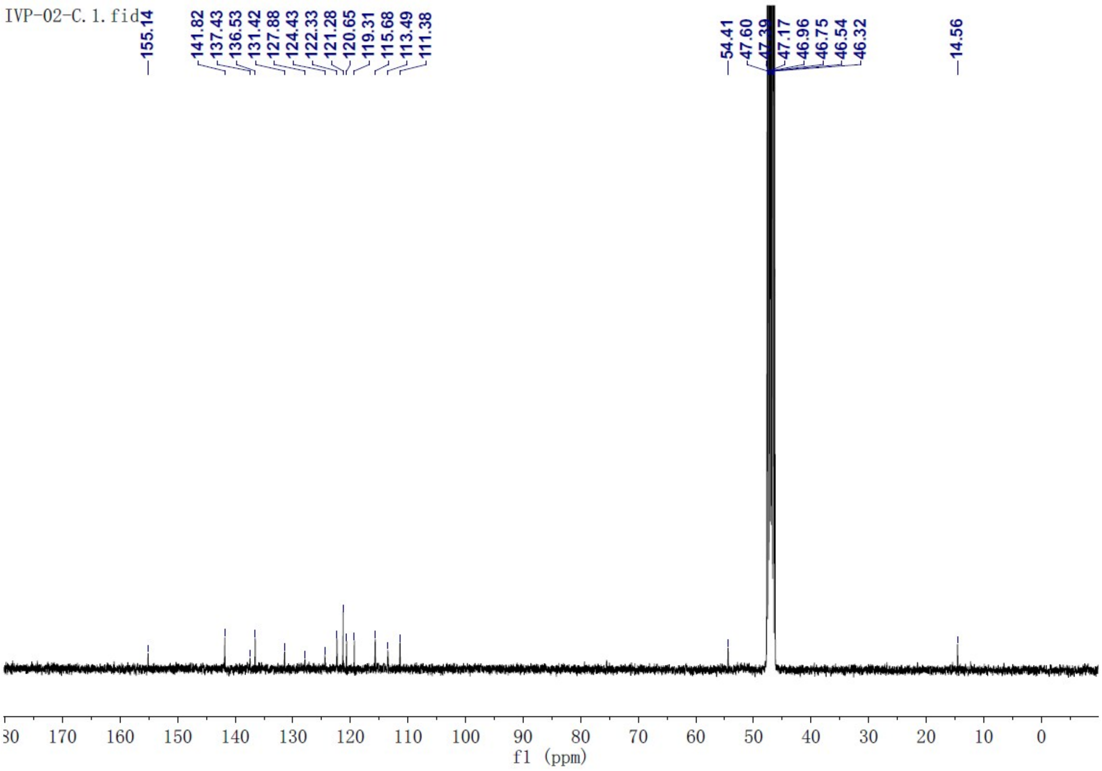

Fig. S26. ^{13}C NMR spectrum of IVP-02 in MeOD-d_4.

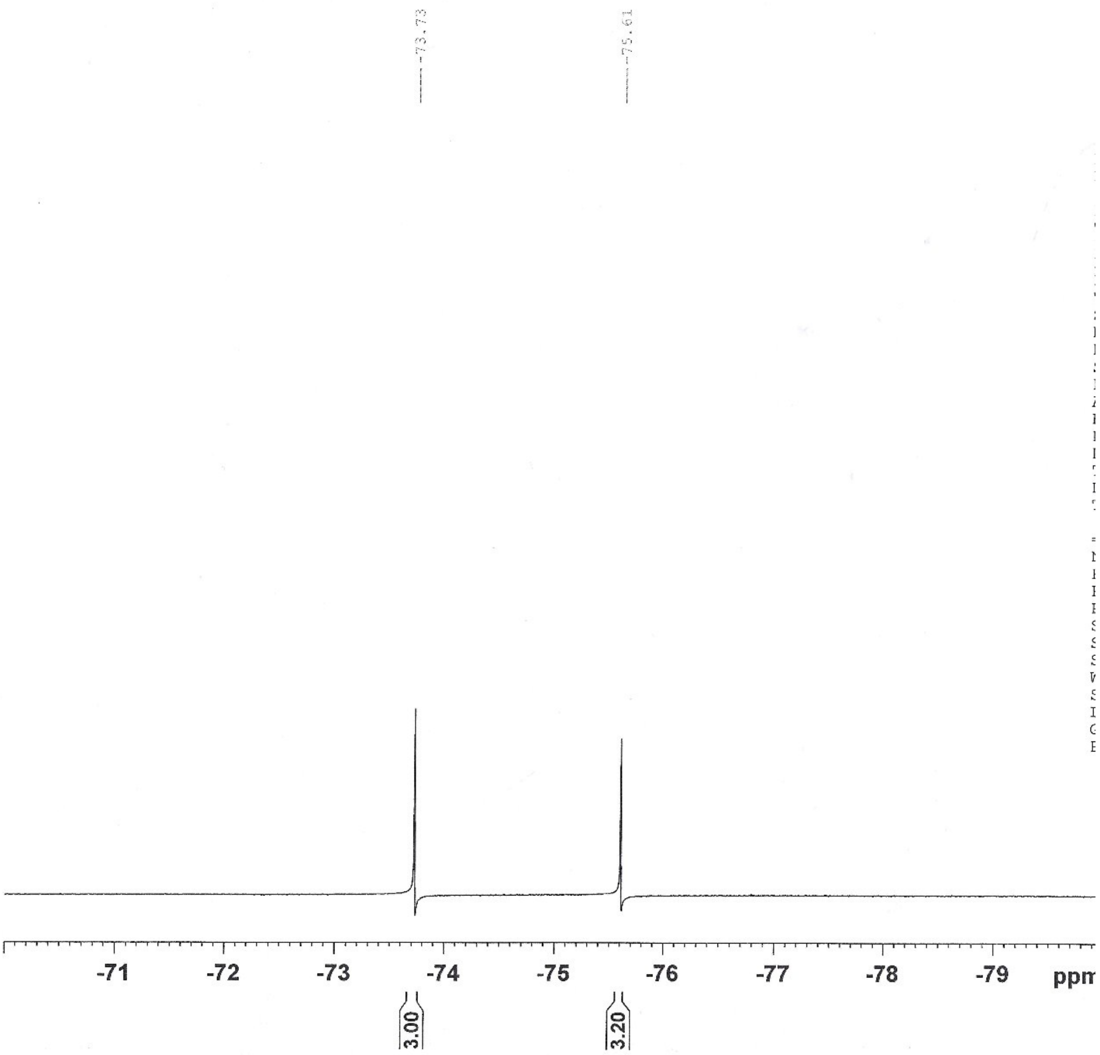

Fig. S27. ^{19}F NMR spectrum of IVP-02 in MeOD-d_4.

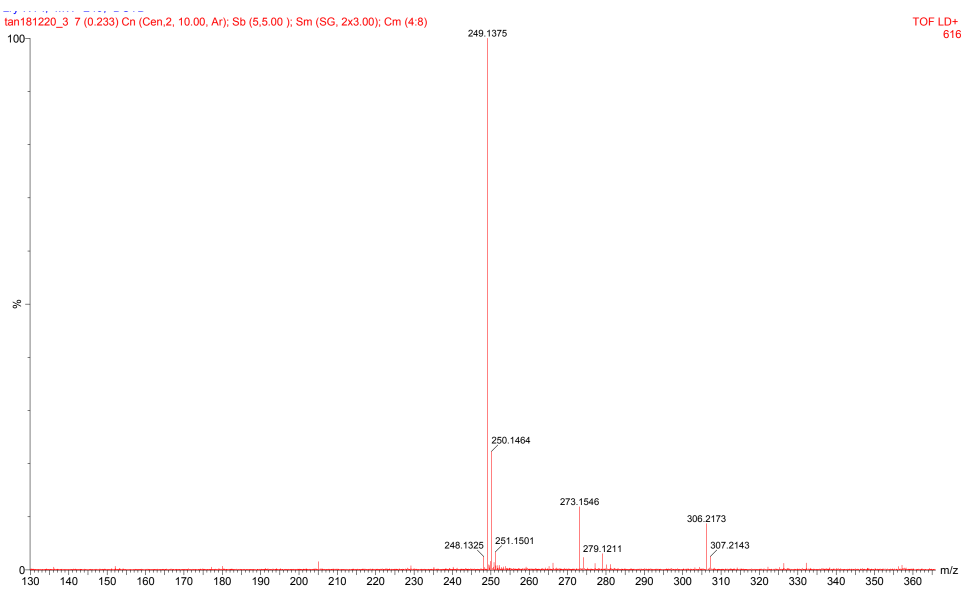

Fig. S28. HRMS of IVP-02.

For IVP-04, ^{1}H NMR (400 MHz, DMSO-d_6), δ (ppm): 11.95 (s, 1H), 8.76 (d, J = 6.60

Hz, 2H), 8.25 (d, J = 16.20 Hz, 1H), 8.14 (dd, J = 11.80 Hz, 7.50 Hz, 3H), 7.97 (s, 1H), 7.52 (dd, J = 7.00 Hz, 1.90 Hz, 1H), 7.35 - 7.21 (m, 3H), 4.41 (t, J = 7.3 Hz, 2H), 1.86 (q, J = 7.40 Hz, 2H), 1.29 (m, 2H), 0.93 (t, J = 7.40 Hz, 3H). ^{13}C NMR (100 MHz, DMSO-d_6), δ (ppm): 154.34, 143.09, 137.36, 136.30, 132.27, 124.66, 122.82, 121.71, 120.05, 116.60, 113.41, 112.45, 58.69, 32.25, 18.62, 13.16. ^{19}F NMR (376 MHz, DMSO-d_6) δ (ppm): -69.18 (s, 3F), -71.07 (s, 3F).

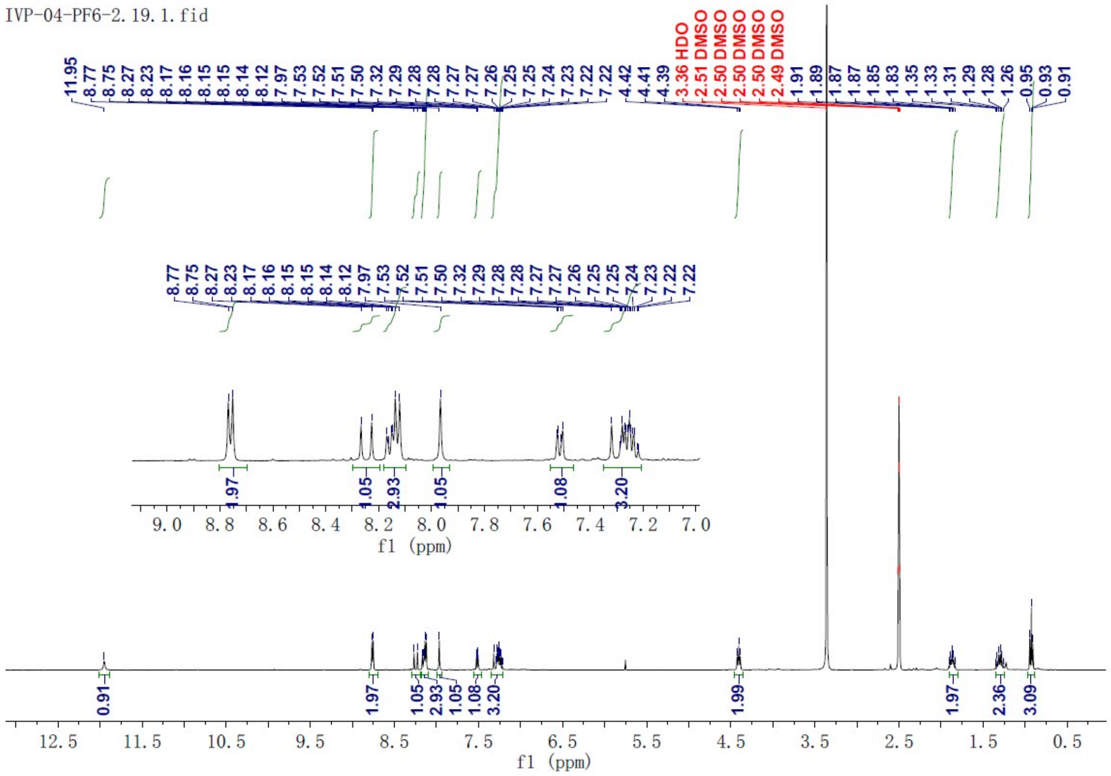

Fig. S29. ^{1}H NMR spectrum of IVP-04 in DMSO-d_6.

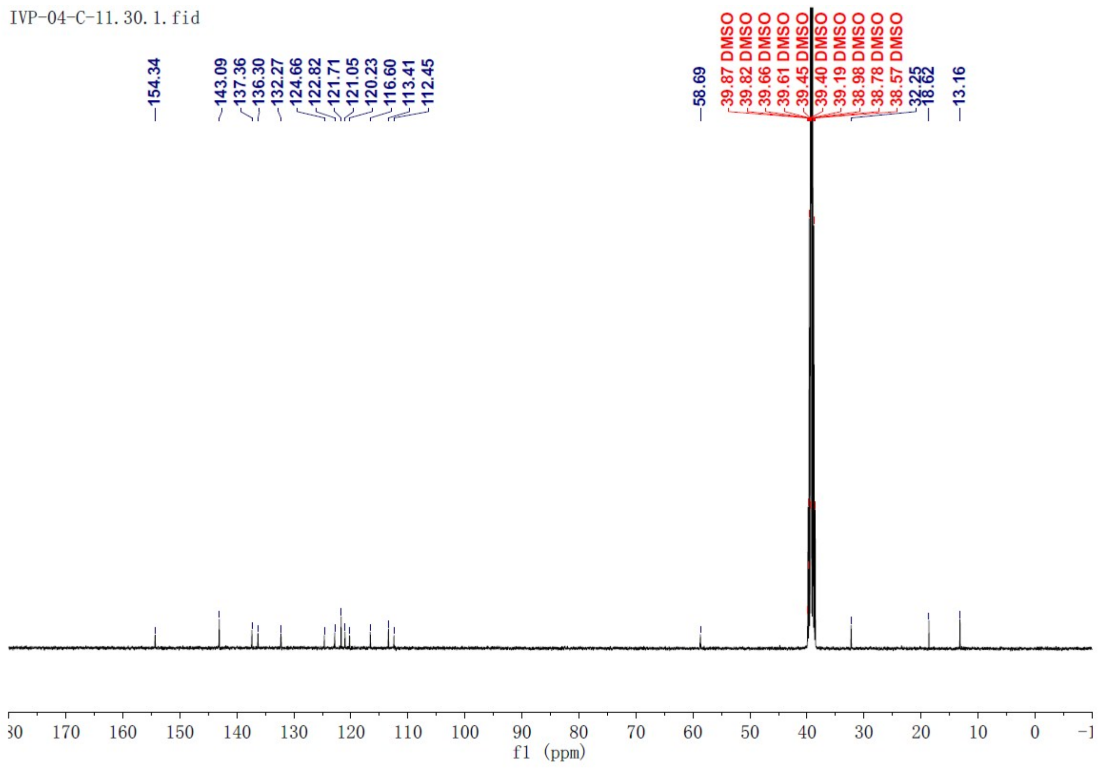

Fig. S30. ^{13}C NMR spectrum of IVP-04 in DMSO-d_6.

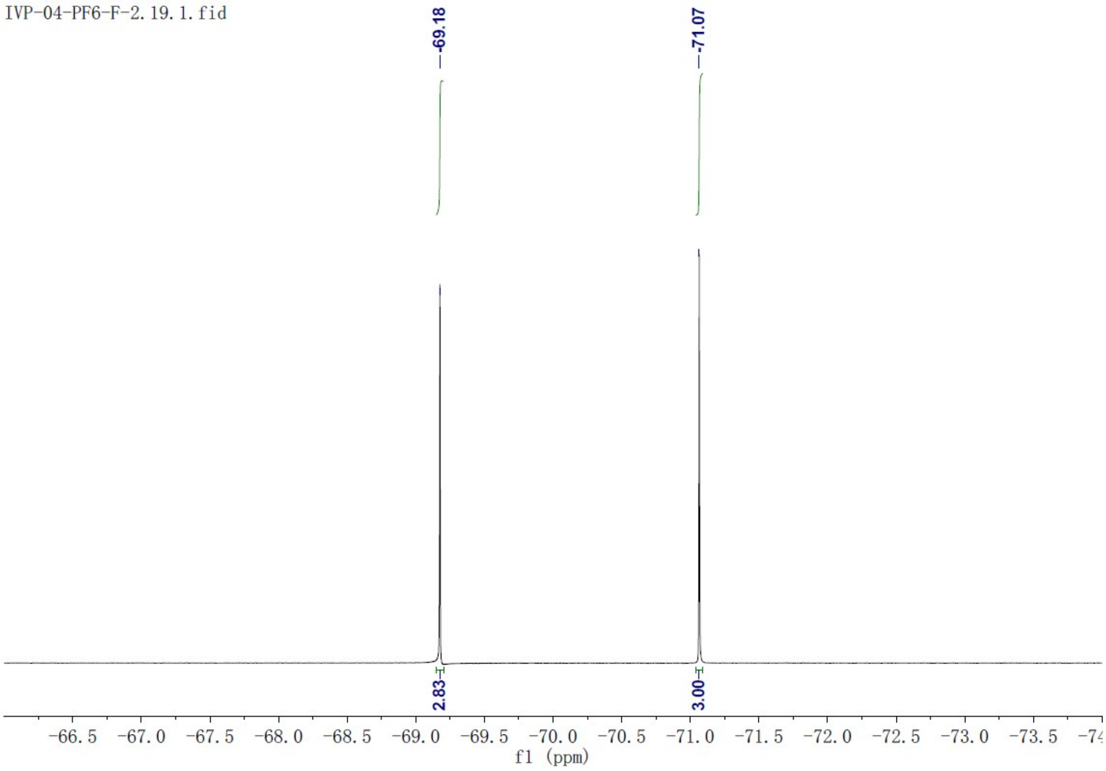

Fig. S31. ^{19}F NMR spectrum of IVP-04 in DMSO-d_6.

For IVP-06, ^{1}H NMR (400 MHz, Acetone-d_6), δ (ppm):11.12 (s, 1H), 8.87-8.81 (m, 2H), 8.35 (d, J = 16.20 Hz, 1H), 8.24 (d, J = 6.60 Hz, 2H), 8.17 (d, J = 7.60 Hz, 1H),

8.02 (d, J = 2.60 Hz, 1H), 7.60 (d, J = 7.80 Hz, 1H), 7.45 (d, J = 16.20 Hz, 1H), 7.37-7.22 (m, 2H), 4.67 (t, J = 7.50 Hz, 2H), 1.47-1.30 (m, 6H), 0.90 (t, J = 6.90 Hz, 3H). ^{13}C NMR (100 MHz, Acetone-d_6), δ (ppm): 154.88, 142.59, 137.40, 136.32, 131.79, 124.60, 122.59, 121.61, 120.89, 120.85, 119.74, 116.36, 113.62, 111.93, 59.44, 30.33, 30.29, 24.88, 21.51, 12.65. ^{19}F NMR (376 MHz, Acetone-d_6), δ (ppm): -71.62 (s, 3F), -73.50 (s, 3F).

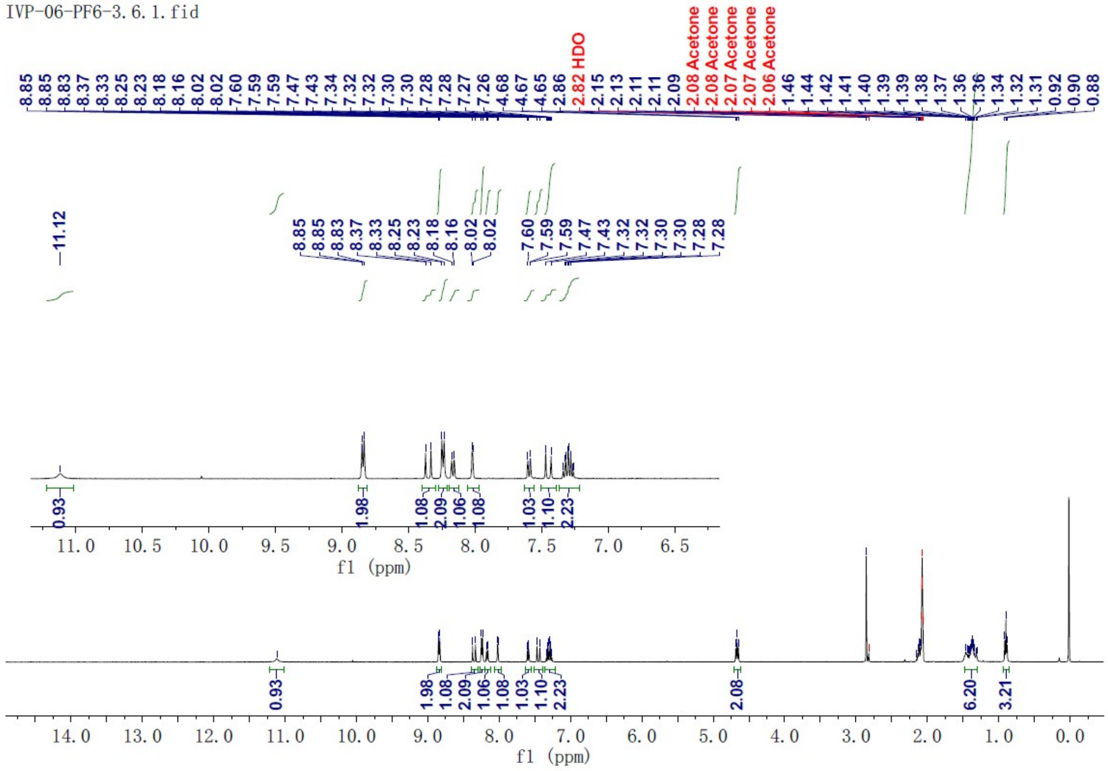

Fig. S32. ^{1}H NMR spectrum of IVP-06 in Acetone-d_6.

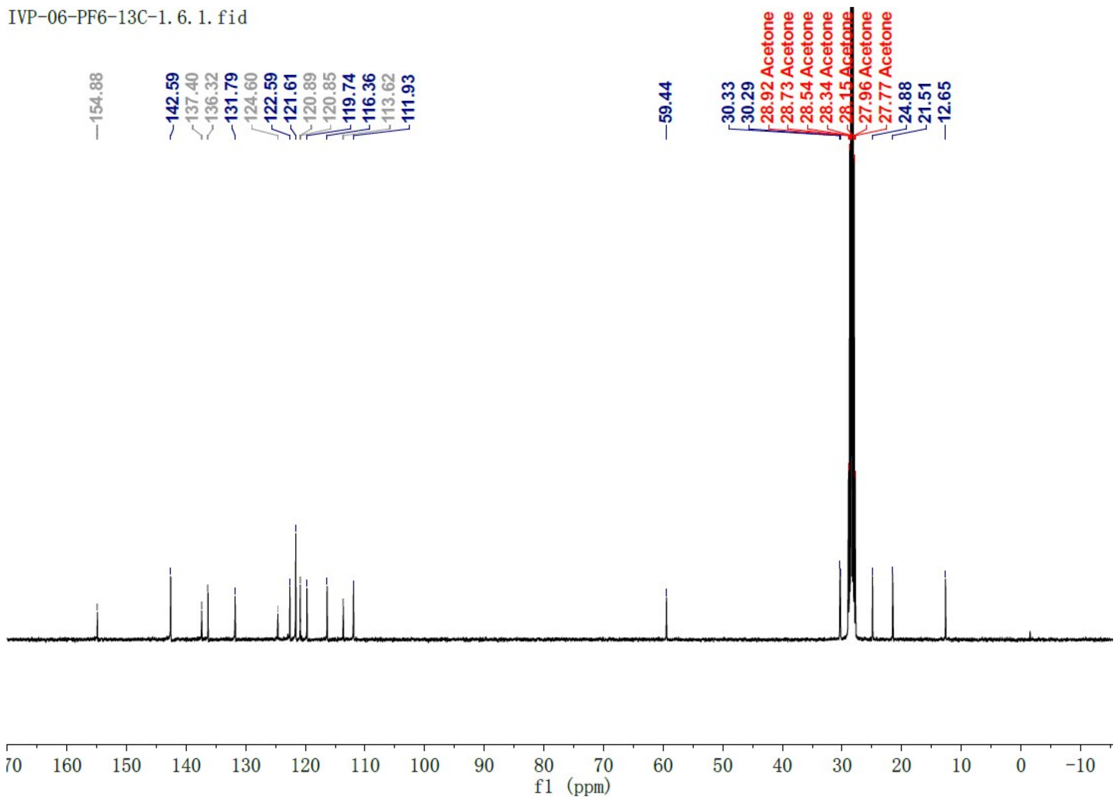

Fig. S33. ^{13}C NMR spectrum of IVP-06 in Acetone-d_6.

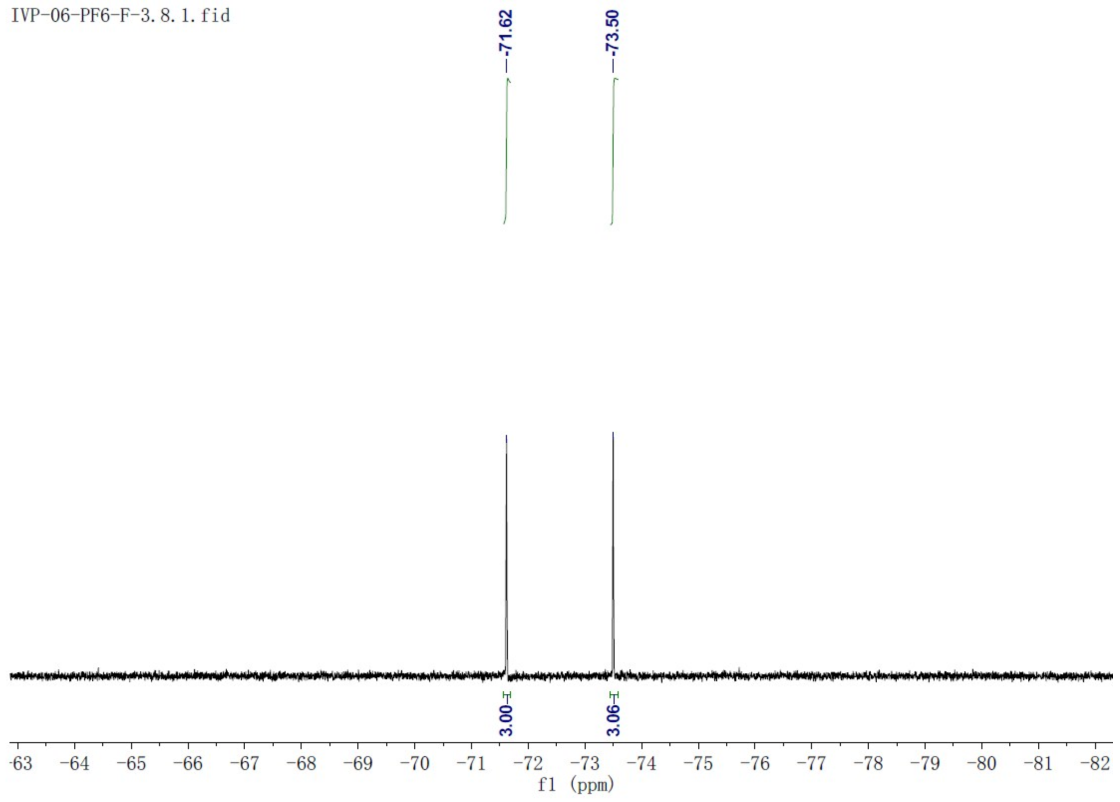

Fig. S34. ^{19}F NMR spectrum of IVP-06 in Acetone-d_6.

For IVP-22, ^{1}H NMR (400 MHz, MeOD-d_4), δ (ppm): 8.55 (d, J = 5.60 Hz, 2H), 8.15

(d, J = 16.10 Hz, 1H), 8.07 (dd, J = 7.30 Hz, 1.50 Hz, 1H), 8.00 (d, J = 7.00 Hz, 2H), 7.87 (s, 1H), 7.53 (d, J = 7.90 Hz, 1H), 7.34-7.17 (m, 3H), 4.45 (q, J = 7.30 Hz, 2H), 4.29 (q, J = 7.30 Hz, 2H), 1.59 (m, 3H), 1.49 (m, 3H). ^{13}C NMR (100 MHz, DMSO-d_6), δ (ppm): 154.21, 142.94, 136.86, 135.59, 133.89, 125.38, 122.81, 121.75, 121.22, 120.48, 116.64, 112.62, 110.84, 54.37, 40.76, 15.98, 15.00. ^{19}F NMR (376 MHz, MeOD-d_4) δ (ppm): -73.84 (s, 3F), -75.72 (s, 3F).

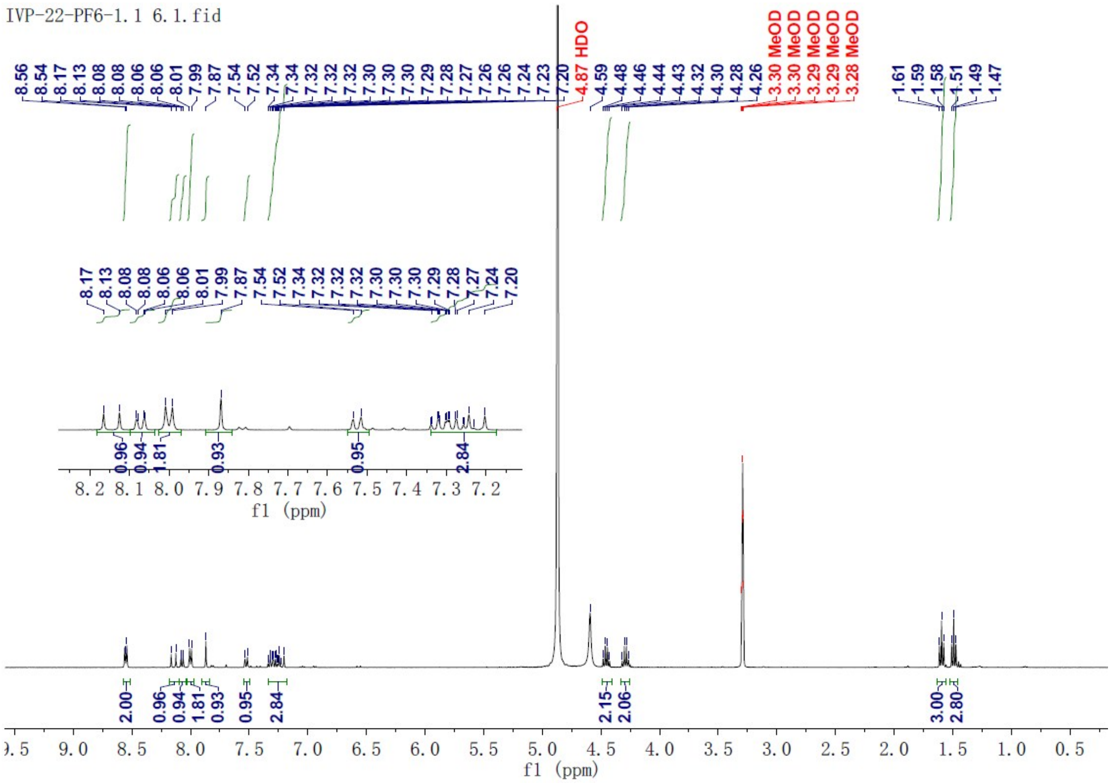

Fig. S35. ^1H NMR spectrum of IVP-22 in MeOD-d_4.

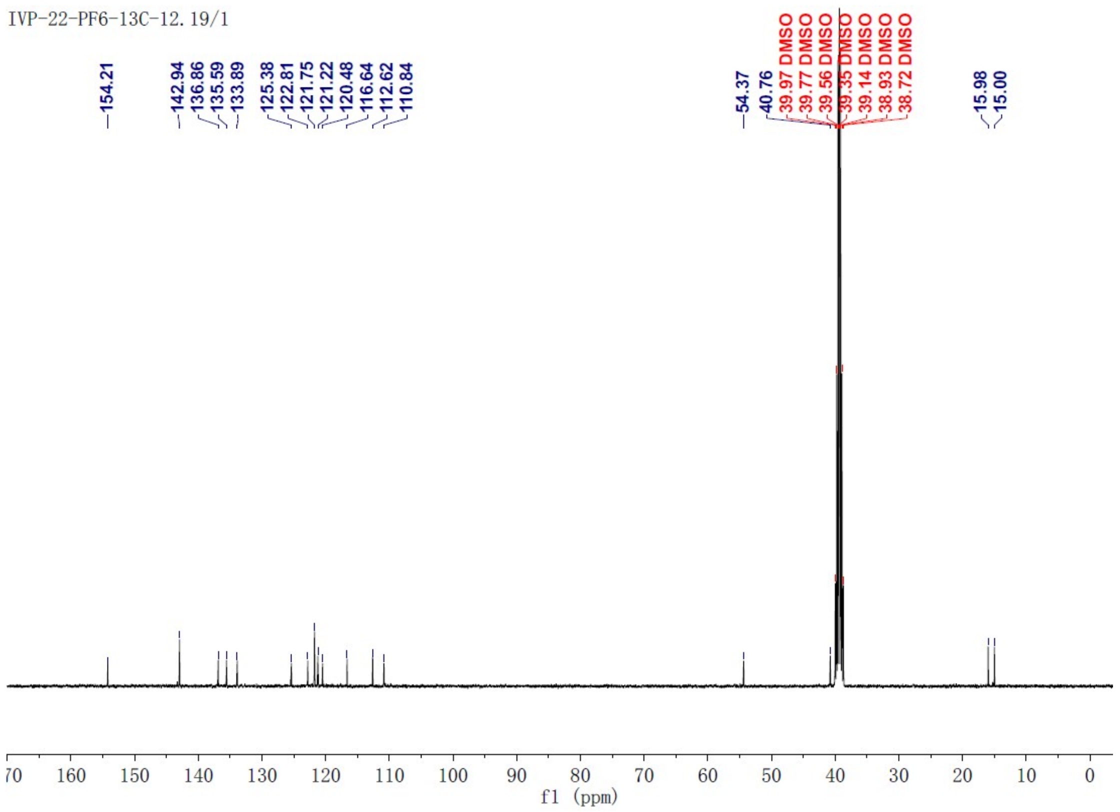

Fig. S36. ^{13}C NMR spectrum of IVP-22 in DMSO-d_6.

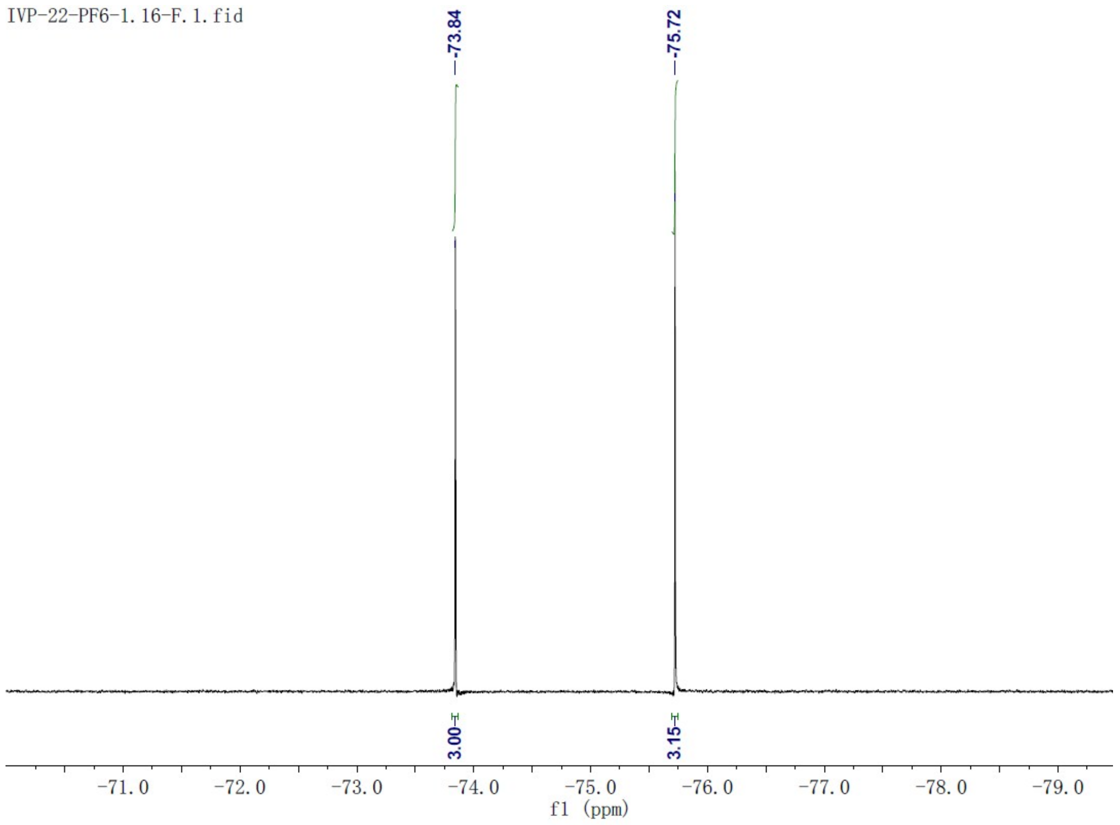

Fig. S37. ^{19}F NMR spectrum of IVP-22 in MeOD-d_4.

For IVP-42, ^{1}H NMR (400 MHz, DMSO-d_6), δ (ppm): 8.77 (d, J = 6.60 Hz, 2H), 8.26-

8.15 (m, 2H), 8.13 (d, J = 6.60 Hz, 2H), 8.01 (s, 1H), 7.63 (d, J = 8.00 Hz, 1H), 7.36-7.24 (m, 3H), 4.44 (q, J = 7.40 Hz, 2H), 4.27 (t, J = 7.00 Hz, 2H), 1.78 (t, J = 7.50 Hz, 2H), 1.51 (t, J = 7.20 Hz, 3H), 1.25 (dd, J = 14.30 Hz, 6.90 Hz, 2H), 0.90 (t, J = 7.30 Hz, 3H). ^{13}C NMR (100 MHz, DMSO-d_6), δ (ppm): 154.20, 142.94, 137.15, 135.57, 134.52, 125.31, 122.81, 121.76, 121.18, 120.46, 116.71, 112.52, 110.95, 54.37, 45.54, 31.39, 19.25, 15.97, 13.34. ^{19}F NMR (376 MHz), δ (ppm): -69.17 (s, 3F), -71.05 (s, 3F).

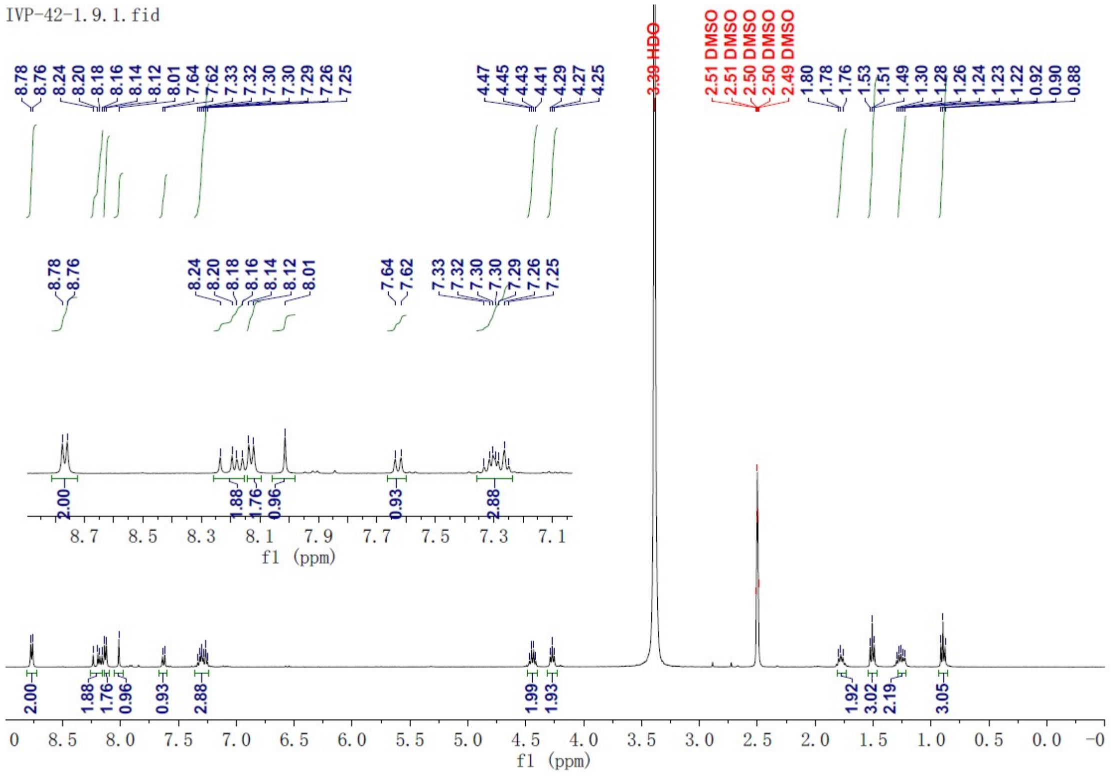

Fig. S38. ^{1}H NMR spectrum of IVP-42 in DMSO-d_6.

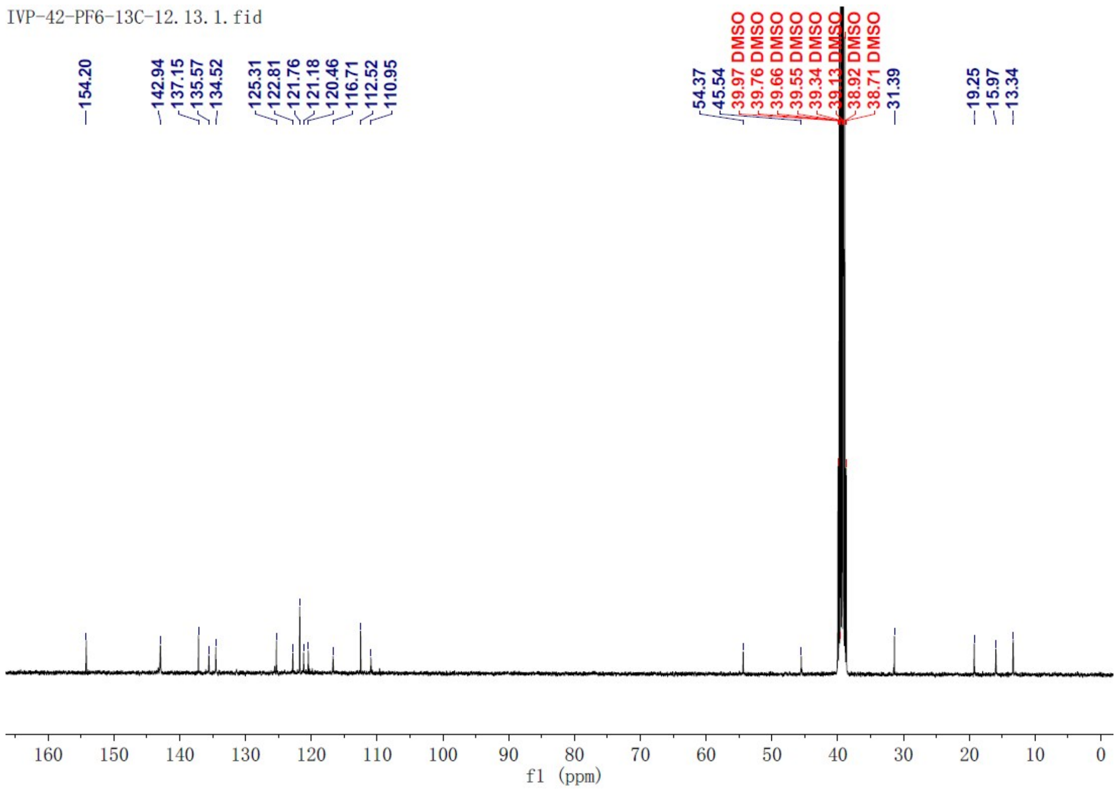

Fig. S39. ^{13}C NMR spectrum of IVP-42 in DMSO-d_6.

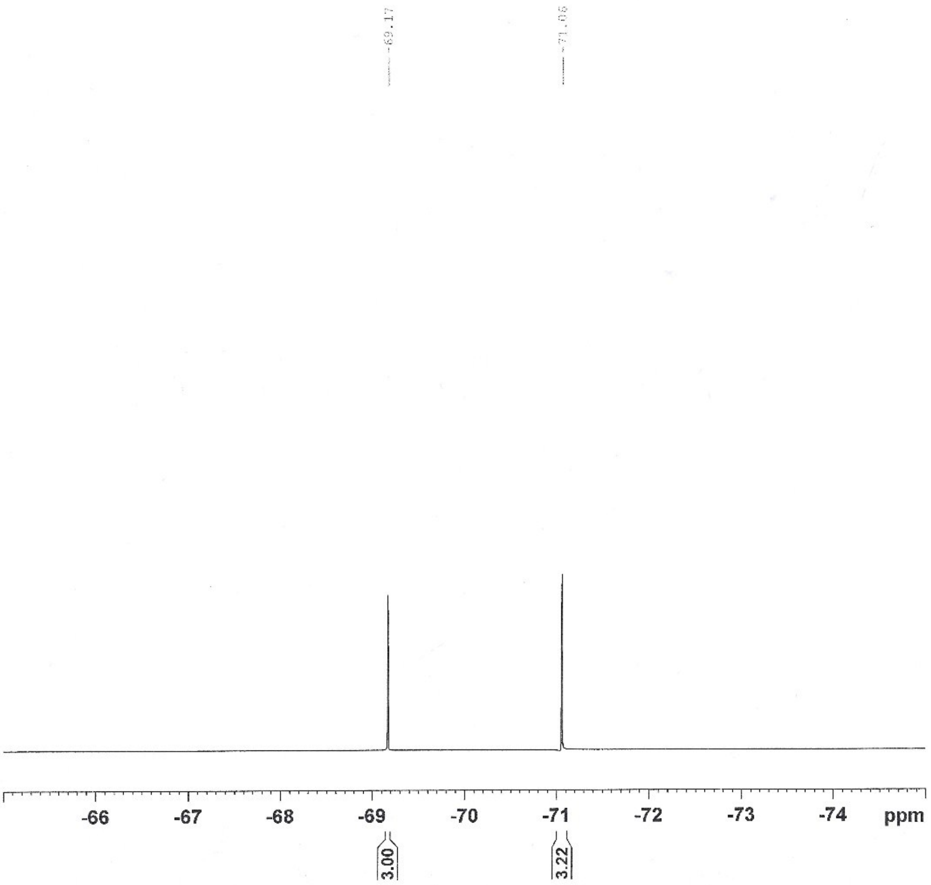

Fig. S40. ^{19}F NMR spectrum of IVP-42 in DMSO-d_6.

For IVP-62, ^{1}H NMR (400 MHz, MeOD-d_4), δ (ppm): 8.49 (d, J = 6.70 Hz, 2H), 8.14-8.01 (m, 2H), 7.94 (d, J = 6.90 Hz, 2H), 7.83 (s, 1H), 7.50 (dd, J = 7.60 Hz, 1.40 Hz, 1H), 7.34-7.24 (m, 2H), 7.17 (d, J = 16.10 Hz, 1H), 4.42 (q, J = 7.40 Hz, 2H), 4.22 (t, J = 7.10 Hz, 2H), 1.86 (t, J = 7.20 Hz, 2H), 1.58 (t, J = 7.30 Hz, 3H), 1.35-1.26 (m, 6H), 0.92-0.80 (m, 3H). ^{13}C NMR (100 MHz, DMSO-d_6), δ (ppm): 154.21, 143.25, 142.93, 137.15, 135.59, 134.53, 125.54, 125.30, 122.81, 121.77, 121.19, 120.46, 119.10, 116.70, 112.52, 110.94, 54.37, 45.82, 30.60, 29.26, 25.65, 21.82, 15.96, 13.69. ^{19}F NMR (376 MHz, MeOD-d_4), δ (ppm): -73.45 (s, 3F), -75.33 (s, 3F).

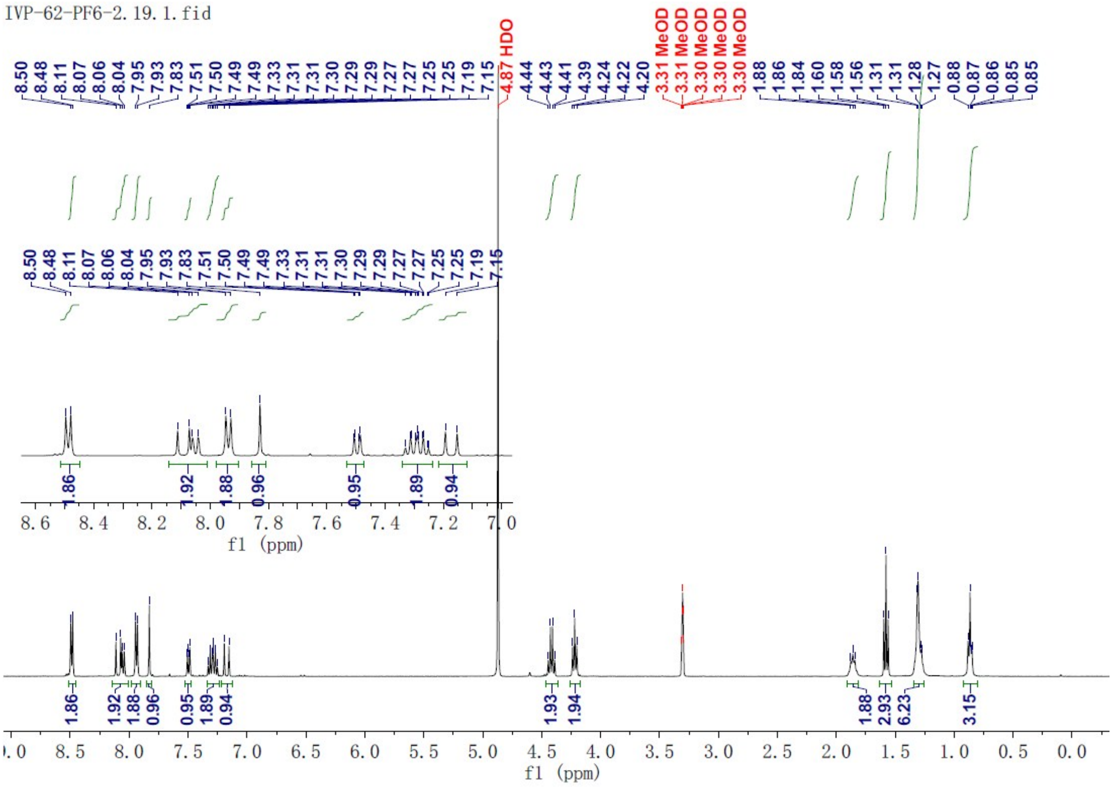

Fig. S41. ^{1}H NMR spectrum of IVP-62 in MeOD -d_4.

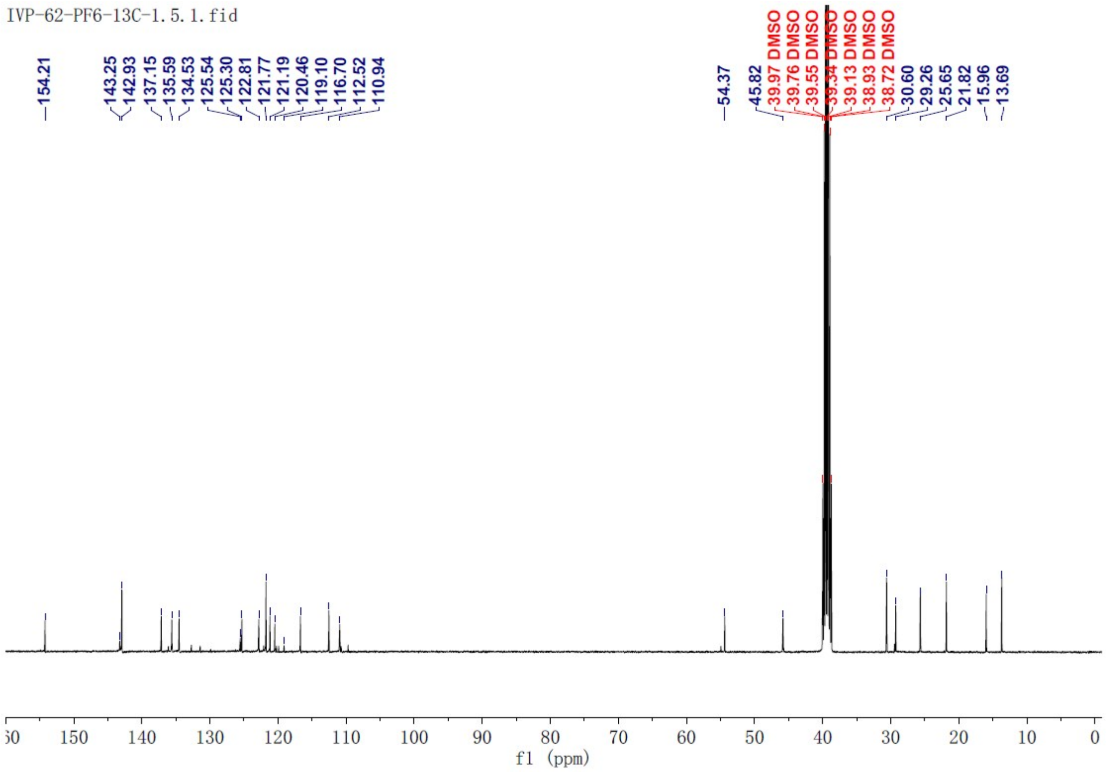

Fig. S42. ^{13}C NMR spectrum of IVP-62 in DMSO-d_6.

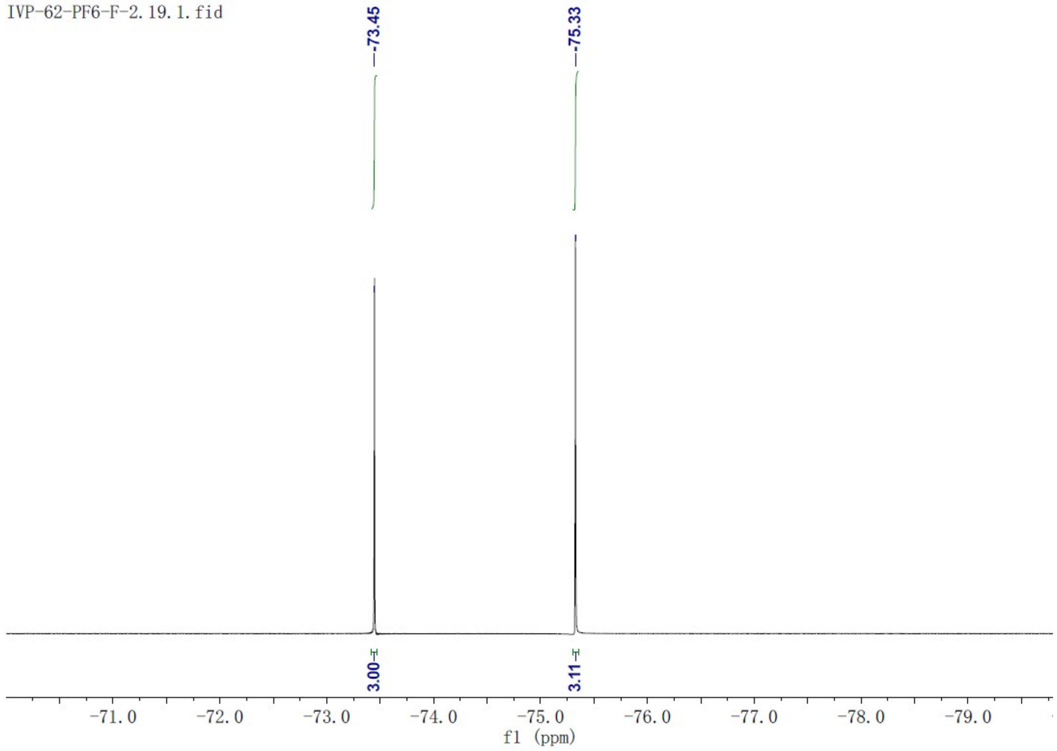

Fig. S43. ^{19}F NMR spectrum of IVP-62 in MeOD -d_4.

7. References

1 R. Zhang, G. Niu, X. Li, L. Guo, H. Zhang, R. Yang, Y. Chen, X. Yu and B. Z. Tang, *Chem. Sci.*, 2019, **10**, 1994–2000.

2 M. Tian, J. Sun, B. Dong and W. Lin, *Angew. Chem. Int. Ed.*, 2018, **57**, 16506–16510.
